# Interactive visualization of nanopore sequencing signal data with *Squigualiser*

**DOI:** 10.1093/bioinformatics/btae501

**Published:** 2024-08-13

**Authors:** Hiruna Samarakoon, Kisaru Liyanage, James M Ferguson, Sri Parameswaran, Hasindu Gamaarachchi, Ira W Deveson

**Affiliations:** School of Computer Science and Engineering, University of New South Wales, Sydney, NSW 2052, Australia; Genomics and Inherited Disease Program, Garvan Institute of Medical Research, Sydney, NSW 2010, Australia; Centre for Population Genomics, Garvan Institute of Medical Research and Murdoch Children’s Research Institute, Sydney, NSW 2010, Australia; School of Computer Science and Engineering, University of New South Wales, Sydney, NSW 2052, Australia; Genomics and Inherited Disease Program, Garvan Institute of Medical Research, Sydney, NSW 2010, Australia; Centre for Population Genomics, Garvan Institute of Medical Research and Murdoch Children’s Research Institute, Sydney, NSW 2010, Australia; Genomics and Inherited Disease Program, Garvan Institute of Medical Research, Sydney, NSW 2010, Australia; Centre for Population Genomics, Garvan Institute of Medical Research and Murdoch Children’s Research Institute, Sydney, NSW 2010, Australia; School of Electrical and Information Engineering, University of Sydney, Sydney, NSW 2008, Australia; School of Computer Science and Engineering, University of New South Wales, Sydney, NSW 2052, Australia; Genomics and Inherited Disease Program, Garvan Institute of Medical Research, Sydney, NSW 2010, Australia; Centre for Population Genomics, Garvan Institute of Medical Research and Murdoch Children’s Research Institute, Sydney, NSW 2010, Australia; Genomics and Inherited Disease Program, Garvan Institute of Medical Research, Sydney, NSW 2010, Australia; Centre for Population Genomics, Garvan Institute of Medical Research and Murdoch Children’s Research Institute, Sydney, NSW 2010, Australia; St Vincent’s Clinical School, Faculty of Medicine, University of New South Wales, Sydney, NSW 2052, Australia

## Abstract

**Motivation:**

Nanopore sequencing current signal data can be ‘basecalled’ into sequence information or analysed directly, with the capacity to identify diverse molecular features, such as DNA/RNA base modifications and secondary structures. However, raw signal data is large and complex, and there is a need for improved visualization strategies to facilitate signal analysis, exploration and tool development.

**Results:**

*Squigualiser* (**Squig**gle vis**ualiser**) is a toolkit for intuitive, interactive visualization of sequence-aligned signal data, which currently supports both DNA and RNA sequencing data from Oxford Nanopore Technologies instruments. *Squigualiser* is compatible with a wide range of alternative signal-alignment software packages and enables visualization of both signal-to-read and signal-to-reference aligned data at single-base resolution. *Squigualiser* generates an interactive signal browser view (HTML file), in which the user can navigate across a genome/transcriptome region and customize the display. Multiple independent reads are integrated into a ‘signal pileup’ format and different datasets can be displayed as parallel tracks. Although other methods exist, *Squigualiser* provides the community with a software package purpose-built for raw signal data visualization, incorporating a range of new and existing features into a unified platform.

**Availability and implementation:**

*Squigualiser* is an open-source package under an MIT licence: https://github.com/hiruna72/squigualiser. The software was developed using Python 3.8 and can be installed with pip or bioconda or executed directly using prebuilt binaries provided with each release.

## 1 Introduction

Nanopore sequencing enables direct analysis of native DNA, RNA, and protein molecules, with countless potential applications across the life sciences (Wang *et al.* 2021). Devices from Oxford Nanopore Technologies (ONT) measure the displacement of ionic current as a DNA or RNA molecule passes through a nanoscale protein pore. The device records time-series current signal data—commonly referred to as ‘squiggle’ data—which can be ‘basecalled’ into sequence reads and/or analysed directly (Wang *et al.* 2021). This signal data is shaped by any molecular feature that influences the flow of ionic current through a pore occupied by a DNA/RNA molecule and/or the rate at which the molecule moves through the pore (the translocation speed). As a result, nanopore sequencing is amenable to detection of diverse ‘modified’ DNA (Simpson *et al.* 2017, Zhang *et al.* 2023) or RNA (Jain *et al.* 2022) nucleotides, DNA damage (An *et al.* 2015), RNA secondary structures (Bizuayehu *et al.* 2022, Stephenson *et al.* 2022), or other features beyond the primary nucleotide sequence. Early-stage demonstrations of protein sequencing further show the suitability of nanopore signal data for determining amino acid sequence and post-translational modifications (Martin-Baniandres *et al.* 2023).

However, nanopore signal data is large, complex and unfamiliar to many in the genomics community. Furthermore, there is currently a lack of purpose-built software for intuitive signal data visualization to assist users in analysing and interpreting signal data, and developing signal-level analysis methods. While various signal alignment or signal analysis packages, such as *Poretools* (Loman and Quinlan 2014), *PoRe* (Watson *et al.* 2015), and ONT’s *Remora* (*Tombo*) contain sub-tools for visualization, these generate static plots with relatively crude signal-to-sequence alignment, and multiple data tracks cannot be displayed in parallel for comparative analysis. The recently released signal aligner *Uncalled4* (Kovaka *et al.* 2024) can be used to generate a semi-interactive signal-to-reference alignment plot with single-base resolution. However, *Uncalled4* does not have capacity to display multiple aligned reads concurrently, limiting its utility for data exploration. Another standalone package for signal visualization, *Bulkvis* (Payne *et al.* 2019), is effective for visualizing raw bulk-FAST5 files but similarly lacks support for signal-to-reference alignment and multi-read display.

Given this fragmented landscape, we set out to develop a more unified and sophisticated software package for nanopore signal data visualization, which would capture useful aspects from existing tools as well as a range of new features. *Squigualiser* (**Squig**gle vis**ualiser**) builds upon existing methodology for signal-to-sequence alignment in order to anchor raw signal data points to their corresponding positions within basecalled reads or within a reference genome/transcriptome sequence. A new encoding technique (the *ss tag*) enables efficient, flexible representation of signal alignments and normalizes outputs from alternative alignment tools. A new method for *k*-mer-to-base shift correction addresses ambiguity in signal alignments to enable visualization of genetic variants, modified bases, or other features, at single-base resolution. *Squigualiser* processes raw or pre-aligned signal data to generate an interactive browser view, in which multiple independent reads and/or datasets can be explored concurrently.

## 2 Visualization framework

*Squigualiser* is a toolkit for visualizing nanopore signal data, which currently supports both DNA and RNA sequencing data from ONT instruments. *Squigualiser* provides the necessary tools to integrate raw signal data, basecalled reads, a reference genome/transcriptome sequence and other genomic feature files, into an intuitive visual format. The software generates an interactive browser view (HTML format) capturing a specified set of reads and/or reference region, which can be displayed and navigated in a web browser (Google Chrome recommended). A pre-built example is provided in Supplementary_File_1.html and a brief instructional video is available at the following link: https://youtu.be/kClYH4KpOjk.

**Figure 1.**
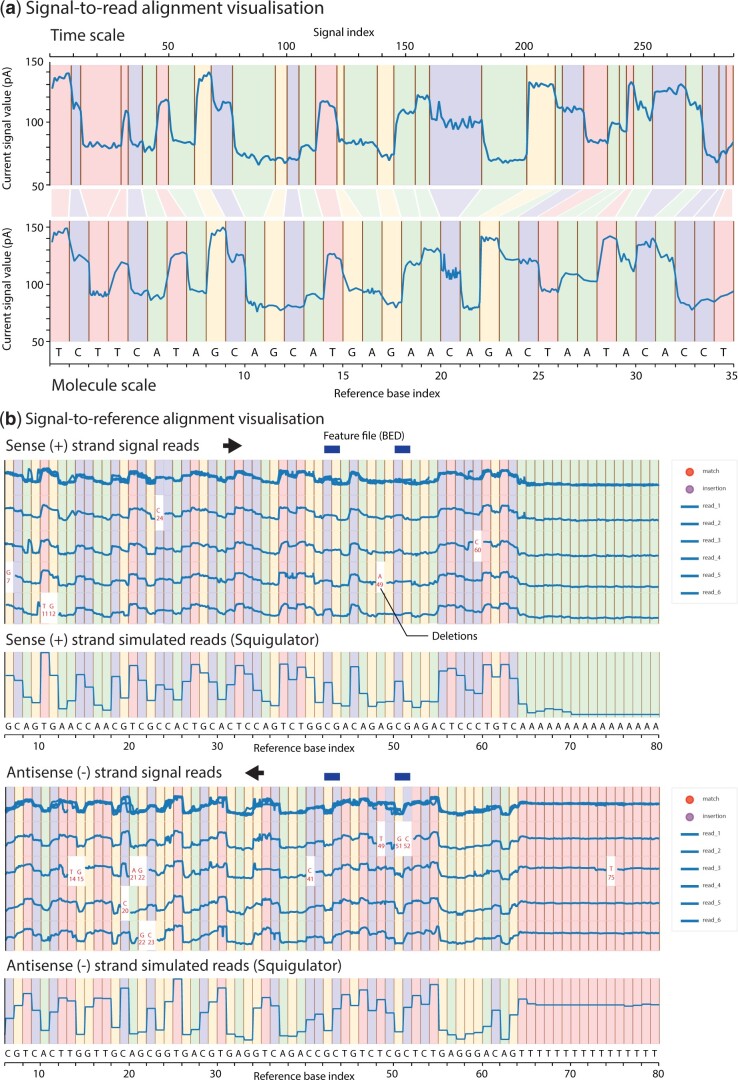
Visualizing sequence-aligned nanopore signal data with *Squigualiser*. (a) Exported images from *Squigualiser* signal viewer show signal-to-read alignments for a ∼35 nucleotide subsequence within a single ONT read. Note images are exported in SVG format and have been partially edited for presentation. In the upper image, aligned signal data is displayed in ‘time-scale’, meaning signal values are spaced uniformly. In the lower image, the same data is displayed in ‘molecule-scale’, wherein signal values are dynamically re-packed such that their aligned nucleotides are displayed with equal width, regardless of how many signal values are aligned to a given base. (b) Exported images show signal-to-reference alignments for a ∼80 nucleotide reference genome region, highlighting various features of the *Squigualiser* visualization strategy. By using molecule-scale, multiple independent reads can be overlaid in a consistent pileup despite variable translocation speeds within and between reads. Reads may be selected/deselected using the control panel (right), to determine which are actively displayed in the pileup. Reads aligned to the antisense (−) strand in DNA sequencing data are shown separately, and in reverse signal orientation (reverse-complement molecule-scale) so that complementary sense/antisense bases are aligned. Simulated signal data from *Squigulator* may be displayed as a parallel track/s to assist signal interpretation.

To provide the molecular context for signal interpretation, *Squigualiser* displays raw signal data points in alignment with their corresponding nucleotides within a basecalled read (signal-to-read) or a reference genome/transcriptome sequence (signal-to-reference). Values for an individual nanopore read are plotted as a string of consecutive points against a vertical axis specifying the measured current (in pico-amps) and a horizontal axis representing the passage of time as the molecule was sequenced (Fig. 1a). The horizontal axis is partitioned and coloured according to the underlying bases (A, C, G, T[U]) in the read/reference sequence. Because translocation speed is not constant, the number of signal data points per nucleotide varies within and between reads. To account for this, when viewing signal-to-read data, the horizontal axis may be alternatively displayed such that the distance between consecutive signal values is equal (time-scale) or dynamically re-packed such that each nucleotide has equal width (molecule-scale; Fig. 1a).

Signal-to-reference alignment enables a user to inspect data from multiple independent reads and/or datasets corresponding to the same genome or transcriptome locus. To facilitate this, *Squigualiser* provides a signal ‘pileup’ view, where individual reads are stacked vertically, aligned in molecule-scale with the underlying reference sequence, and all reads are overlaid in a single track above (Fig. 1b). The user can interactively select/deselect reads to determine which are actively displayed within the pileup. For DNA sequencing data, signal alignments to the antisense (−) strand are displayed in reverse orientation, so that data points are aligned with their corresponding base in the genome reference, and sense (+), and antisense (−) reads are organized in separate pileup tracks (Fig. 1b). Multiple independent datasets may also be displayed as separate, parallel tracks, similar to the layout in popular genome browsers, such as Integrative Genomics Viewer (IGV) (Robinson *et al.* 2011). A coordinate annotation file (BED format) may be displayed, in order to identify relevant features within the reference (e.g. methylated CpG sites; Fig. 1b).

Our recent tool for nanopore signal data simulation, *Squigulator* (Gamaarachchi *et al.* 2024), can be used to generate and display a simulated data track within *Squigualiser*, based on the underlying read/reference sequence. This ‘expected’ data track provides useful context, with deviations in the accompanying experimental data being potentially indicative of sequence variants, modified bases or other features (Fig. 1b). Insertions and deletions commonly occur within signal-to-reference alignments, due to a combination of basecalling and/or alignment errors and true genetic variants. To accommodate this, *Squigualiser* leaves empty space within aligned signal reads at the positions of deleted nucleotides and labels the identity of missing bases (Fig. 1b). Signal data points corresponding to inserted bases are stacked into a single point at the border of the preceding reference nucleotide and can be revealed by enabling ‘show signal points’ from the legend.

## 3 Materials and methods

### 3.1 Implementation

*Squigualiser* is developed in Python 3.8 and relies on several essential dependencies: *Bokeh* library to create interactive base-signal alignment plots; *pyslow*, *readpaf*, *pyfaidx*, *pyfastx*, and *pysam* for reading input file formats; *numpy* for data handling; and, *matplotlib* and *seaborn* to generate density plots in PDF format. *Squigualiser* can be installed via the pip package management tool. We also provide prebuilt binaries for linux-x86-64 and macOS-arm64 architectures with each new version. These were constructed using the snake-charming technique described in (Ferguson *et al.* 2022) and contain the Python interpreter along with all the dependencies, allowing the user to simply extract and execute. *Squigualiser’s* usage and implementation are described in further detail in the Supplementary Note S2.

### 3.2 Signal alignment methods

Signal-to-sequence alignment is central to this visualization framework and several methodological innovations were required to streamline and optimize this process. *Squigualiser* reads and writes signal alignments in BAM and PAF formats familiar to the genomics community, but customized for signal data. For example, we developed a new auxiliary tag for encoding signal alignments within BAM or PAF format, named the ‘*ss tag*’. Inspired by the CIGAR string from sequence alignment format (Li *et al.* 2009), the *ss tag* encodes signal-to-sequence matches, insertions or deletions of varying lengths in a minimal alphanumeric string and is described in detail in Supplementary Note S1.

The *ss tag* is an efficient encoding method that also serves to normalize the signal alignment representations used by different software. This allows *Squigualiser* to support alignment data generated through a variety of alternative methods, including with *Uncalled4* (Kovaka *et al.* 2024), *f5c resquiggle* (Gamaarachchi *et al.* 2020), *f5c eventalign* (Gamaarachchi *et al.* 2020), *Nanopolish* (Simpson *et al.* 2017) signal projection, and *Sigfish* (Shih *et al.* 2023) dynamic time warping methods. Alternatively, the user may instead directly extract and re-format signal-to-read alignment information from the ONT move table generated during basecalling, using the *Squigualiser reform* subtool. This may also be integrated with basecalled read alignments (BAM format) to generate signal-to-reference alignments, using the *Squigualiser realign* subtool. Different approaches to signal alignment supported by *Squigualiser* are compared within Supplementary Figs S4 and S5 in Supplementary Note S2.

### 3.3 *K*-mer-to-base shift correction

Existing signal-to-read and signal-to-reference alignment methods work by anchoring ‘events’ (step changes in signal values) not to individual nucleotides but to *k*-mers (DNA/RNA sub-sequences of length = *k*). Therefore, there is an arbitrary offset (distance *n*; where *n* < *k*) between a given base and the most appropriate event to represent that base in the aligned signal. This makes it difficult to precisely associate signal events with genetic variants, DNA/RNA modifications, or other features at single-base resolution.

It is known that specific bases have predictable effects on the current level during nanopore sequencing, as determined by their size, shape, charge, etc. (Manrao *et al.* 2012). We reasoned that these effects could be used to calculate and correct the arbitrary offset in signal alignments, for more intuitive visualization. To do so, we identify the most significant base (MSB) within a given *k*-mer (i.e. the single base with the greatest influence on the current level), infer the local signal event with which it is likely associated, and measure the offset between them. Applied globally to a given dataset, this identifies an offset value by which all signal alignments in the dataset should be shifted in order to align all bases with their most relevant signal events. This is protocol-specific; the appropriate offset value differs for different flow-cell versions, basecalling models and/or signal alignment methods, and therefore needs to be re-calculated for a given protocol. *Squigualiser* includes a subtool *calculate_offsets* to identify the appropriate base-shift for a given dataset and, as default option, will automatically calculate and apply appropriate *k*-mer-to-base shift correction to any dataset provided by the user. Supplementary Note S3 describes the development and validation of *Squigualiser’s k*-mer-to-base shift correction in detail.

### 3.4 Methods for fast data retrieval and visualization

*Squigualiser* can generate a pileup for signal alignments to a specific region of large genomes such as the human genome in seconds. This is achieved by using indexable input formats for fast random access, including FASTA/FASTQ for sequence data, BAM or bgzip compressed PAF for signal-to-sequence mapping, and BLOW5 (Gamaarachchi *et al.* 2022) for signal data (see Supplementary Note S2). Signal data stored in ONT’s native FAST5 or POD5 file formats can be easily converted to BLOW5 format using our tool *bluecrab* (https://github.com/Psy-Fer/blue-crab) and indexed using *slow5tools* (Samarakoon *et al.* 2023), prior to visualizing with *Squigualiser*.

### 3.5 Example use case

There are many foreseeable use-cases for the signal data visualization frameworks provided by *Squigualiser*. To provide two simple examples, we used *Squigualiser* to visually inspect a set of modified DNA and RNA bases in ONT datasets (see Supplementary Notes S4 and S5) Modified DNA or RNA bases, such as the epigenetic regulator 5-methylcytosine (5mC), are associated with distortions of ONT signal data relative to their corresponding canonical bases, which are the basis for their detection. However, modifications are diverse, subtle and may be challenging to detect (Xu and Seki 2020). Given the capacity to visualize multiple independent signal reads and/or datasets (e.g. case versus control), aligned to their appropriate position in the reference genome/transcriptome with single-base resolution, *Squigualiser* is well-suited for visual inspection of candidate modifications.

Supplementary Note S4 demonstrates how *Squigualiser* can be used to visualize the distinct signal patterns between 5mC-modified versus unmodified DNA reads aligned to a partially methylated CpG site. Supplementary Note S5 explores the visualization of synthetic RNA molecules produced pseudouridine (ψ) in place of canonical U bases (Gunter *et al.* 2023).

## Supplementary Material

btae501_Supplementary_Materials

## Data Availability

The HG002 dataset sequenced on an ONT R10.4.1 PromethION flowcell used for the figures is publicly available on the NCBI Sequence Read Archive (SRR23215366). The synthetic unmodified and modified RNA datasets used in Supplementary Note S5 are also publicly available on NCBI SRA (SRR22888949, SRR22888950).
