# Supplementary material for: Interactive visualization of nanopore sequencing signal data with Squigualiser
Source: Bioinformatics. 2024 Aug 13;40(8):btae501. doi: 10.1093/bioinformatics/btae501 (PMC11335371; doi:10.1093/bioinformatics/btae501)
Supplement: btae501_Supplementary_Materials [file btae501_supplementary_materials.zip › SupplementaryMaterials/Supplementary Note 1.pdf]

Supplementary Note 1: Signal Alignment Formats/Tags

Hiruna Samarakoon, Kisaru Liyanage, James M. Ferguson, Sri Parameswaran, Hasindu Gamaarachchi, Ira W. Deveson

July 1, 2024

Note: Read this document sequentially. Skipping sections without prior context is not recommended, as the content is not reiterated.

This document explains the formats and tags supported by squigualiser. Signal-to-read alignment feature in Squigualiser requires the signal alignment information to be in PAF format explained in section 1.1. The signal-to-reference alignment feature can take either the PAF format (section 2.1) or SAM/BAM format (section 2.2) and a brief comparison of different encoding methods is followed (section 3). In both PAF and SAM format, the signal alignment is encoded using an auxiliary tag called the *Signal alignment String (ss)*. In PAF format, the start and end coordinates of the signal mapping are in relevant PAF columns, whereas in SAM format these coordinates are in the *Signal Information (si)* tag. Currently, the latest available versions of f5c and squigulator can directly output these formats compatible with squigualiser. We encourage developers working on signal alignment methods to adhere to this specification when generating outputs, ensuring wider compatibility with tools such as squigualiser.

1 Signal-to-read alignment

1.1 PAF format

PAF format supported by Squigualiser (this format is inspired by UNCALLED) contains the columns described in Table 1. In this signal-to-read alignment context, the query is the raw-signal and the target is the basecalled-read.

Table 1: Columns in PAF format for signal-to-read alignment

Col	Type	Name	Description
1	string	read_id	Read identifier name
2	int	len_raw_signal	Raw signal length (number of samples)
3	int	start_raw	Raw signal start index (0-based; BED-like; closed)
4	int	end_raw	Raw signal end index (0-based; BED-like; open)
5	char	strand	Relative strand: "+" or "-"
6	string	read_id	Same as column 1
7	int	len_kmer	base-called sequence length (no. of k-mers)
8	int	start_kmer	k-mer start index on basecalled sequence (0-based; see note below)
9	int	end_kmer	k-mer end index on basecalled sequence (0-based; see note below)
10	int	matches	Number of k-mers matched on basecalled sequence
11	int	len_block	Same as column 7
12	int	mapq	Mapping quality (0-255; 255 for missing)

Conventions: For DNA reads, column 8 is a closed coordinate (inclusive) and column 9 is an open coordinate (not-inclusive); and, column 8 coordinate is always smaller than column 9. However, this is different in direct-RNA reads because the sequencing of direct-RNA happens in the reverse direction (3'->5') and therefore the raw signal is also in the reverse direction. The basecalled read output by basecallers is however in the correct direction (5'->3'). Thus, For RNA reads, the column 8 coordinate will be larger than that of column 9.

Column 9 is a closed coordinate (inclusive) while column 8 is an open coordinate (not-inclusive) in this case, contrary to DNA. Columns 10,11 and 12 are not used by Squigualiser and thus are not finalised.

Auxiliary tags: Auxiliary tags supported by squigualiser are described in Table 2, where the *ss* tag is mandatory. The `sh` and `sc` tag values can be used to scale the raw signal to the pore model. This can be done as: `scaled pA current values = (pA - sh) / sc`, where, `pA = (raw_signal + offset) * range / digitisation))`. The *ss* tag is described in detail below in section 1.1.1

Table 2: Auxilliary tags in PAF format

Tag	Type	Description
sc	f	Post alignment recalibrated scale parameter
sh	f	Post alignment recalibrated shift parameter
ss	Z	signal alignment string in format

1.1.1 ss tag

ss tag is a custom encoding that compacts the signal-base alignment. It can be thought of as an extended version CIGAR string that accommodates for signal alignment needs. This *ss* string was inspired by the *--sam* option in Nanopolish for eventalign.

Consider the example `8,5,4,8I4,3D4,5,` for DNA. This means 8 signal samples map to the starting base of the sequence; the next 5 samples to the next base, the next 4 samples to the next base; 8 next samples are missing a mapping in the basecalled read (insertion to reference); 4 samples map to the next base; 3 bases in the basecalled read have no corresponding signal samples (deletion); 4 samples map to the next base; and 5 samples map to the next base. Note that the start indexes of read and signal are the absolute values in columns 8 and 3 respectively above. the *ss* string is relative to this.

The '`,`', '`D`' and '`I`' can be though of as three different operations. The '`,`' after a number means step one base in the basecall reference, while step number of samples preceding the '`,`' in the raw signal. The '`D`' after a number means step number of bases preceding the '`D`' in the basecalled read and no stepping in the raw signal. The '`I`' after a number means step number of samples preceding the '`I`' in the raw signal and no stepping in the basecalled read.

1.1.2 DNA Example

To make things further clear, given in Figure 1 is an illustration for an alignment of DNA raw-signal to a basecalled read.

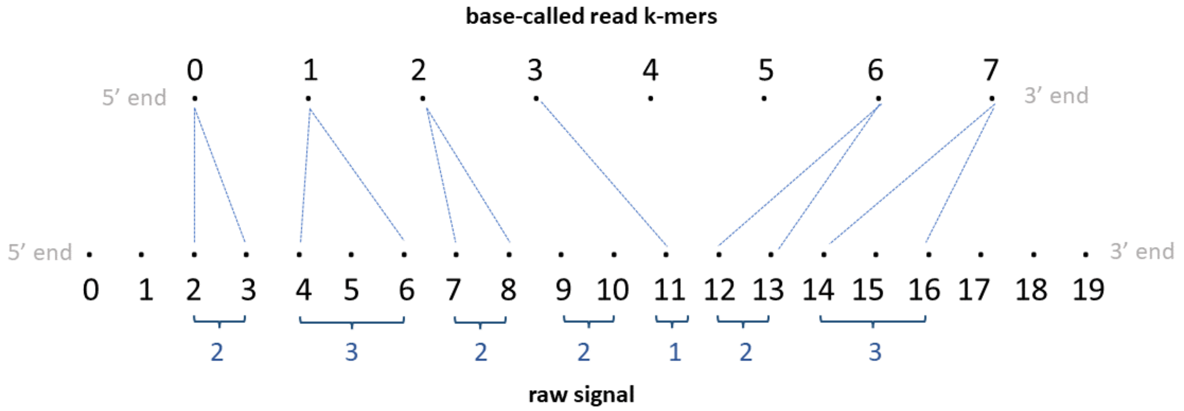

Figure 1: DNA example for signal-to-read alignment

Each dot in the top sequence in Figure 1, represents a k-mer and the number above each dot is the corresponding k-mer index. If the basecalled read is `ACGGTAACTATAC` and assuming the k-mer size in the k-mer model is 6, the 0th k-mer is `ACGGTA`, 1st k-mer is `CGGTAA` ... and the 7th k-mer is `CTATAC`. Each dot in

the bottom sequence in the illustration represent a raw-signal sample and the number below each dot is the corresponding signal index.

The PAF output for the example in Figure 1 will look like in Table 3 (the header is not present in the actual output).

Table 3: DNA signal-to-read alignment example in PAF format

read_id	len_raw_signal	start_raw	end_raw	strand	read_id	len_kmer	start_kmer	end_kmer	matches	len_block	mapq	
rid0	20	2	17	+	rid0	8	0	8	6	8	255	ss:Z:2,3,2,2I1,2D2,3,

1.1.3 direct-RNA Example

Now see the illustration in Figure 2 for direct-RNA.

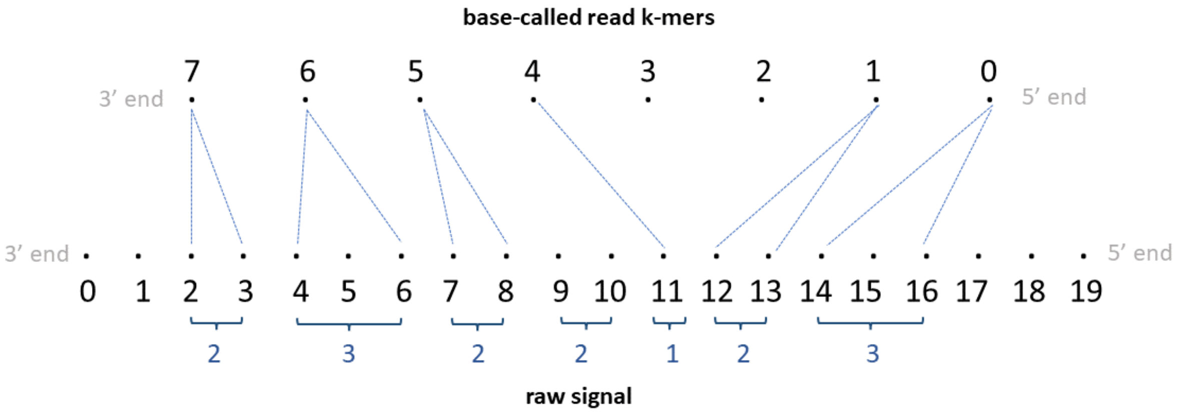

Figure 2: RNA example for signal-to-read alignment

Note that the RNA is sequenced from 3'->5' end, so the raw signal is 3'->5' direction. As the basecaller outputs the basecalled read in 5'->3' direction, the basecalled read is reversed to be 3'->5' in the illustration (note: indices in illustration denote the actual index in the basecalled read in 5'->3' direction). If the basecalled read in 5'->3' direction is `ACGGUAACUAUA` and assuming the k-mer size in the k-mer model is 5, the 0th k-mer is `ACGGU`, 1st k-mer is `CGGUA` . . . and the 7th k-mer is `CUAUA`.

The PAF output for the example RNA in Figure 2 will look like in Table 4.

Table 4: RNA signal-to-read alignment example in PAF format

read_id	len_raw_signal	start_raw	end_raw	strand	read_id	len_kmer	start_kmer	end_kmer	matches	len_block	mapq	
rid0	20	2	17	+	rid0	8	8	0	6	8	255	ss:Z:2,3,2,2I1,2D2,3,

1.1.4 C code snippet to parse ss tag

A C code snippet that converts the value in the ss tag (a readable code that is not optimised) is given below:

```c
#include <stdio.h>
#include <stdlib.h>
#include <ctype.h>

#define MAX_LEN_KMER 20000

int main(){

    //paf fields
    char *ss="2,3,2,2I1,2D2,3,";
    int start_raw=2; int end_raw=17; int len_raw_signal=20;
    int start_kmer=0; int end_kmer=8; int len_kmer=8;

    // Raw signal start index for the corresponding k-mer and Raw signal end index for the corresponding k-mer
    int st_raw_idx[MAX_LEN_KMER]; int end_raw_idx[MAX_LEN_KMER];

    //intialise to -1
    for(int i=0; i<MAX_LEN_KMER; i++){ st_raw_idx[i]=end_raw_idx[i]=-1; }

    int st_k = start_kmer; int end_k = end_kmer; //if DNA, start k-kmer index is start_kmer column in paf and end k-kmer index is end_kmer column in paf
    int8_t rna = start_kmer > end_kmer ? 1 : 0; //if RNA start_kmer>end_kmer in paf
    if(rna){ st_k = end_kmer; end_k = start_kmer; } //if RNA, start k-kmer index is end_kmer column in paf and end k-kmer index is start_kmer column in paf

    int i_k = st_k; int i_raw = start_raw; //current k-mer index and current raw signal index

    //buffer for storing digits preceding each operation and its index
    char buff[11]; int i_buff=0;

    while(*ss){

        if(*ss==',' || *ss=='I' || *ss=='D'){
            if(i_buff <= 0){ fprintf(stderr,"Bad ss: Preceding digit missing\n"); exit(1); }//if nothing in buff

            buff[i_buff]=0; //null terminate buff
            int num = atoi(buff);
            if(num < 0){ fprintf(stderr,"Bad ss: Cannot have negative numbers\n"); exit(1); }
            i_buff=0; buff[0]=0; //reset buff

            if(*ss=='I'){ //if an insertion, current raw signal index is incremented by num
                i_raw += num;
            } else if(*ss=='D'){ //if an deletion, current k-mer index is incremented by num
                i_k += num;
            } else if (*ss==','){ //if a mapping, increment accordingly and set raw signal indices for the current k-mer
                end_raw_idx[i_k] = i_raw; i_raw += num;
                st_raw_idx[i_k] = i_raw; i_k++;
            }
        } else {
            if(!isdigit(*ss)){ fprintf(stderr,"Bad ss: A non-digit found when expected a digit\n"); exit(1); }
            buff[i_buff++]=*ss;
        }
        ss++;
    }

    if(i_raw!=end_raw){ fprintf(stderr,"Bad ss: Signal end mismatch\n"); exit(1); } //current raw signal index should be equal to end_raw
    if(i_k!=end_k){ fprintf(stderr,"Bad ss: Kmer end mismatch\n"); exit(1); } //current k-mer index should be equal to end_k

    for(int i=st_k; i<end_k; i++){
```

```
        if(end_raw_idx[i]==-1){
            if(st_raw_idx[i] != -1) { fprintf(stderr,"Bad ss: This shoud not have happened\n"); exit(1); }//if st_raw_idx[i] is -1, then end_raw_idx[i] should also be -1
            printf("%d\t.\t.\n", rna ? len_kmer-i-1 : i);
        }else {
            printf("%d\t%d\t%d\n", rna ? len_kmer-i-1 : i, end_raw_idx[i], st_raw_idx[i]);
        }
    }
}
```

2 Signal-to-reference alignment

2.1 PAF format

The PAF format supported by Squigualiser for the signal-to-reference feature is similar to PAF output explained in section 1.1, with major difference being that the "basecalled read" is now the "refererence sequence". Assuming that the reader is well familiarised with the PAF output explained in section 1.1, that information is not repeated here, instead only a summary is given. The PAF columns are explained in Table 5. Unlike in section 1.1, the strand column (column 5) can be now both '+' and '-'. The query is the raw-signal and the target is the reference.

Table 5: Columns in PAF format for signal-to-reference alignment

Col	Type	Name	Description
1	string	read_id	Read identifier name
2	int	len_raw_signal	Raw signal length (number of samples)
3	int	start_raw	Raw signal start index (0-based; BED-like; closed)
4	int	end_raw	Raw signal end index (0-based; BED-like; open)
5	char	strand	Relative strand: "+" or "-"
6	string	read_id	Reference sequence name
7	int	len_kmer	Reference sequence length (no. of k-mers)
8	int	start_kmer	k-mer start index on reference sequence (0-based; see note below)
9	int	end_kmer	k-mer end index on sequence sequence (0-based; see note below)
10	int	matches	Number of k-mers matched on reference sequence
11	int	len_block	Number of k-mers on the mapped segment on reference sequence
12	int	mapq	Mapping quality (0-255; 255 for missing)

The conventions (paragraph conventions) and auxiliary tags (paragraph auxiliary tags and section 1.1.1 described before are applicable here. Some examples are given below.

2.1.1 DNA examples

DNA Positive strand example: Assume we have a read signal named rid0 of 1000 signal samples, mapped to a reference contig named ctg0 of 35 bases Assume a k-mer size of 6. We have a total of 30 k-mers in the reference. Assume the signal-reference alignment looks like in Figure 3. Assume that the 12-24th bases (0-index; bed-like) in this contig are `TTGATGGTGGAA`. Thus, 12th kmer is TTGATG, 13th k-mer is TGATGG, 14th k-mer is GATGGT, .. and the 18th k-mer is GTGGAA.

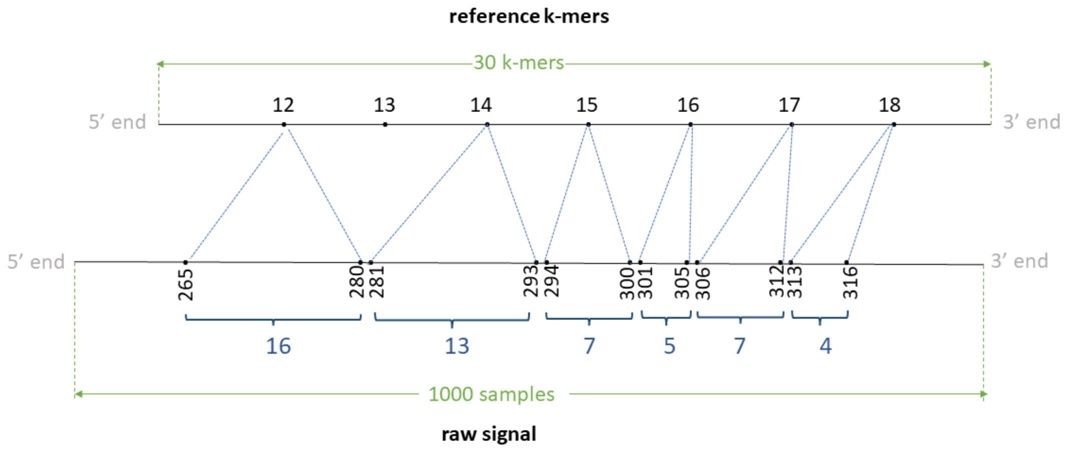

Figure 3: DNA example for signal-to-reference alignment (mapped to positive strand)

The PAF output from eventalign will look like in Table 6 (the header is not present in the actual output).

Table 6: DNA signal-to-reference alignment example in PAF format (mapped to positive strand)

read_id	len_raw_signal	start_raw	end_raw	strand	ref_id	len_kmer	start_kmer	end_kmer	matches	len_block	mapq	
rid0	1000	265	317	+	ctg0	30	12	19	6	7	255	ss:Z:16,1D13,7,5,7,4,

DNA Negative strand example: Assume we have a read signal named rid1 of 1000 signal samples, mapped to a reference contig named ctg0 of 35 bases Assume a k-mer size of 6. We have a total of 30 k-mers in the reference. Assume the signal-reference alignment looks like in Figure 4 (note: indices in illustration denote the actual index in the + strand of the reference genome in 5'->3' direction).

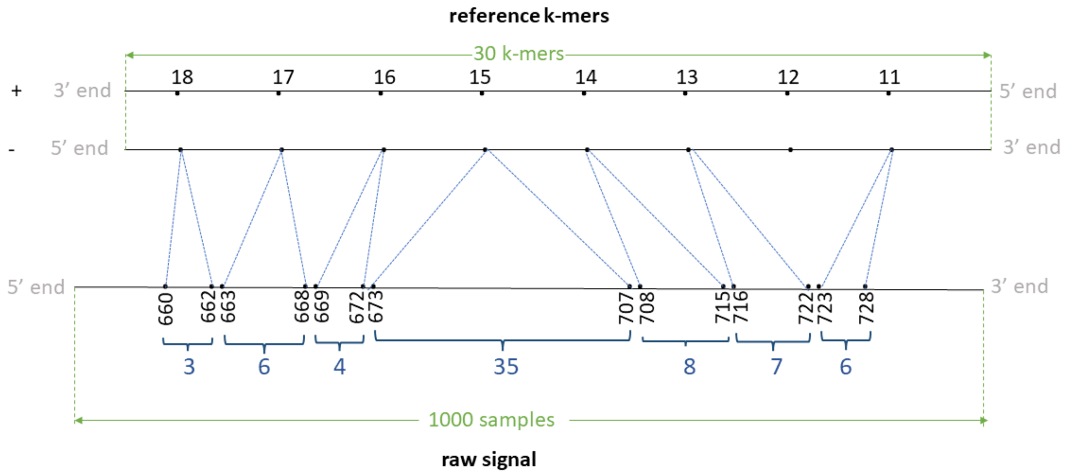

Figure 4: DNA example for signal-to-reference alignment (mapped to negative strand)

Assume that the 11-24th bases (0-index; bed-like) in this contig are ATTGATGGTGGAA. Thus, 11th kmer is ATTGAT, 12th k-mer is TTGATG, 13th k-mer is TGATGG, .. 17th k-mer is GGTGGA and the 18th k-mer is GTGGAA. Th negative strand is like:

```
5' ATTGATGGTGGAA 3' + strand
   |||||||||||||
3' TAACTACCACCTT 5' - strand
```

The Reverse complement is thus TTCCACCATCAAT. The 11th k-mer ATTGAT in the + strand relates to ATCAAT in the - strand, 12th k-mer TTGATG relates to CATCAA, 13th k-mer TGATGG relates to CCATC ,. . . , 17th k-mer GGTGGA relates to TCCACC and 18th k-mer GTGGAA relates to TTCCAC.

The PAF output from eventalign will look like in Table 7 (the header is not present in the actual output).

Table 7: DNA signal-to-reference alignment example in PAF format (mapped to negative strand)

read_id	len_raw_signal	start_raw	end_raw	strand	ref_id	len_kmer	start_kmer	end_kmer	matches	len_block	mapq	
rid1	1000	660	729	-	ctg0	30	11	19	7	8	255	ss:Z:3,6,4,35,8,7,1D6,

2.1.2 RNA examples

RNA Positive strand example: Assume we have a read signal named rid0 of 3000 signal samples, mapped to a reference transcript (or can be a ctg in the reference genome) named trn0 of 65 bases. Assume a k-mer size of 5. We have a total of 61 k-mers in the reference.

Assume the signal-reference alignment looks like in the Figure 5. Note that the RNA is sequenced 3'->5' end, so the raw signal is 3'->5' direction. However, as transcripts in the reference are in 5'->3' direction, the transcript is reversed to be 3'->5' in the illustration (note: indices in illustration denote the actual index in the transcript in 5'->3' direction).

Assume that the 45-56th bases (0-index; bed-like) in this transcript in 5'->3' direction is GAGAGCCCTGA. Then, 45th kmer is GAGAG, 46th k-mer is AGAGC, 47th k-mer is GAGCC, .. and the 51st k-mer is CCTGA.

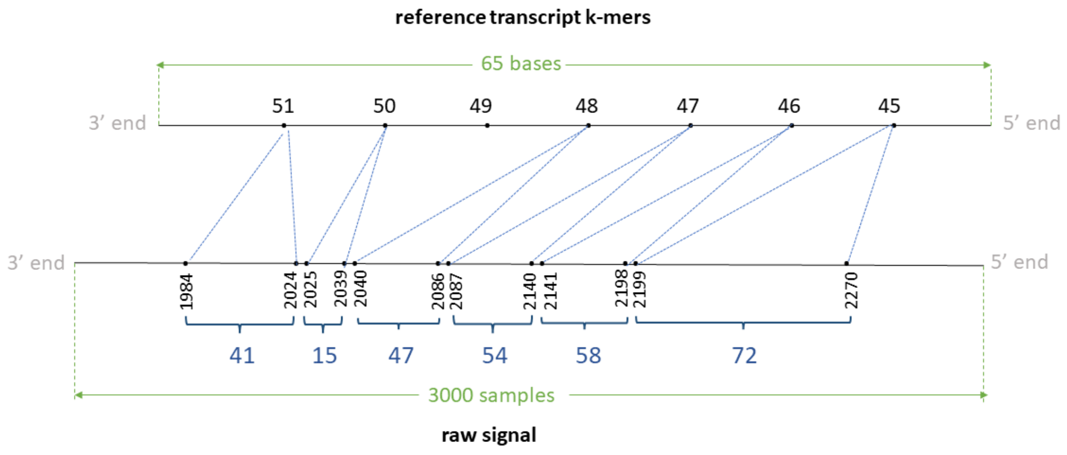

Figure 5: RNA example for signal-to-reference alignment (mapped to positive strand)

The PAF output from eventalign will look like in Table 8 (the header is not present in the actual output):

Table 8: RNA signal-to-reference alignment example in PAF format (mapped to positive strand)

read_id	len_raw_signal	start_raw	end_raw	strand	ref_id	len_kmer	start_kmer	end_kmer	matches	len_block	mapq	
rid0	3000	1984	2271	+	trn0	61	52	45	6	7	255	ss:Z:41,15,1D47,54,58,72,

Note that start_kmer and end_kmer are otherway round compared to DNA.

RNA Negative strand example Assume we have a read signal named rid1 of 500 signal samples, mapped to a reference contig named ctg1 of 20 bases. Assume a k-mer size of 5. We have a total of 24 k-mers in the reference. Assume the signal-reference alignment looks like in Figure 6 (note: indices in illustration denote the actual index in the + strand of the reference genome in 5'->3' direction).

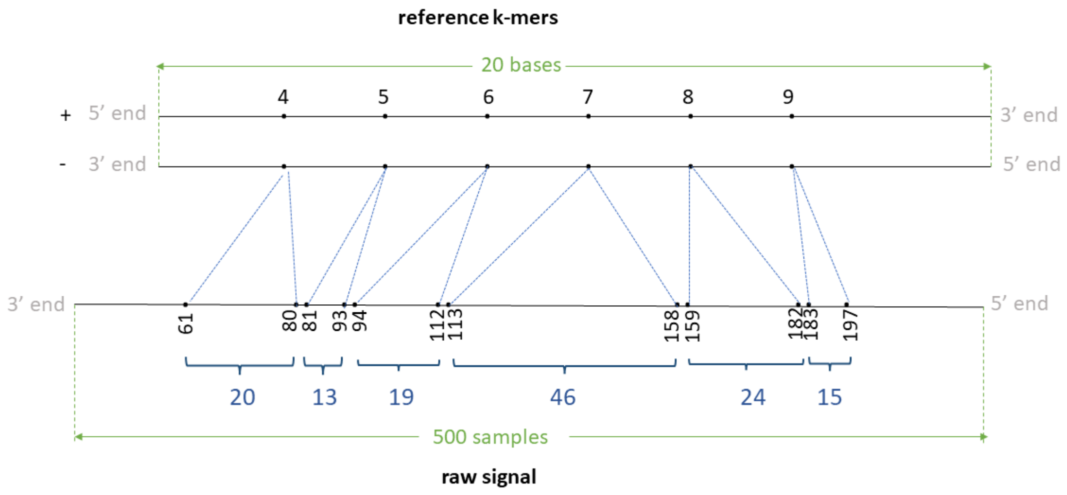

Figure 6: RNA example for signal-to-reference alignment (mapped to negative strand)

Assume that the 4-13th bases (0-index; bed-like) in this contig are AAATGGCTGA. Thus, 4th kmer is AAATG, 5th k-mer is AATGG, .. 8th k-mer is GGCTG and the 9th k-mer is GCTGA. The negative strand is like:

```
5' AAATGGCTGA 3' + strand
   ||||||||||
3' TTTACCGACT 5' - strand
```

The Reverse complement is thus TCAGCCATTT. The 4th k-mer AAATG in the + strand relates to CATTT in the - strand, 5th k-mer AATGG relates to CCATT ,. . . , 8th k-mer GGCTG relates to CAGCC and 9th k-mer GCTGA relates to TCAGC.

The PAF output from eventalign will look like in Table 9 (the header is not present in the actual output):

Table 9: RNA signal-to-reference alignment example in PAF format (mapped to negative strand)

**read_id**	**len_raw_signal**	**start_raw**	**end_raw**	**strand**	**ref_id**	**len_kmer**	**start_kmer**	**end_kmer**	**matches**	**len_block**	**mapq**	
rid1	500	61	198	-	ctg1	24	10	4	6	6	255	ss:Z:20,13,19,46,24,15,

2.2 SAM format

Auxiliary tags supported by squigualiser in SAM format are described in Table 10, where the *si* and *ss* tags are mandatory. The `sh` and `sc` tag values can be used to scale the raw signal to the pore model in the same way stated in paragraph auxiliary tags for PAF format. The *si* tag contains four comma separated values *start_raw*, *end_raw*, *start_kmer* and *end_kmer*, respectively. Those values are the same as the columns 3,4,8 and 9 in the PAF format explained in Table 5 for PAF format. The *ss* tag is same as described in detail in section 1.1.1.

Table 10: Auxilliary tags in SAM format

**Tag**	**Type**	**Description**
sc	f	Post alignment recalibrated scale parameter
sh	f	Post alignment recalibrated shift parameter
si	Z	signal information tag containing coordinates associated with the ss tag
ss	Z	signal alignment string in format described under section 1.1.1

3 Comparison of signal-to-sequence alignment encoding formats

The important reason for developing the *ss* tag, rather than using CIGAR format, is that CIGAR encoding cannot store signal alignments at single-base resolution. For example, CIGAR uses '3M' to encode 3 aligned bases matching the reference sequence. However, this does not encode the number of signal points per base. Hence, we need an operator (,) that moves per base. For example, the '3M' read-to-reference mapping above can have a signal-to-reference mapping as 10,5,15, which means 10 samples mapped to the first base, 5 to the second and 15 bases to the third. In this way, the *ss* tag adds additional functionality to handle signal alignment with high resolution.

An additional analysis comparing the size / compression ratio of our new *ss* tag to other possible encodings for signal alignments is outlined in the table 11. Possible options here include *mv* tag from ONT basecallers, for signal-to-read alignments, the *ul* tag from UNCALLED4 for signal-to-reference encoding, as well as a basic TSV format used by nanopolish. We note that the TSV to SAM comparison is not a like-for-like comparison, as the different formats include somewhat different information and have different capabilities. For example, the TSV format stores additional information as the contig, reference k-mer, model k-mer etc.

Table 11: Comparison of signal-to-sequence alignment encoding formats

**signal-to-read alignment encoding**	**size (bytes)**	**size (GB)**	**ratio**
mv tag ASCII	10773227538	10.77	
ss tag ASCII	6449493246	6.45	1.670399073
**signal-to-reference encoding**	**size (bytes)**	**size (GB)**	**ratio**
ul tag ASCII	6130396215	6.13	
ss tag ASCII	6092964456	6.09	1.006143448
**signal-to-reference alignment file sizes**	**size (bytes)**	**size (GB)**	**ratio**
nanopolish/f5c TSV	461075784533	461.08	
f5c SAM	9958472100	9.96	46.29985202
