# Supplementary material for: Interactive visualization of nanopore sequencing signal data with Squigualiser
Source: Bioinformatics. 2024 Aug 13;40(8):btae501. doi: 10.1093/bioinformatics/btae501 (PMC11335371; doi:10.1093/bioinformatics/btae501)
Supplement: btae501_Supplementary_Materials [file btae501_supplementary_materials.zip › SupplementaryMaterials/Supplementary Note 2.pdf]

Supplementary Note 2: *Squigualiser* implementation, methods and usage

Hiruna Samarakoon, Kisaru Liyanage, James M. Ferguson, Sri Parameswaran, Hasindu Gamaarachchi, Ira W. Deveson

July 1, 2024

1 Installation

Squigualiser is developed and tested using Python 3.8 and can be installed via the pip package management tool. Prebuilt binaries (for linux-x86-64 and macOS-arm64 architectures), which can be simply extracted and executed, are released with each version. These pre-built binary packages were constructed using the snake-charming technique described in [1], and contain the Python interpreter along with all the dependencies.

2 Browser view

Squigualiser generates plots as HTML (or SVG) files that can be visualised on a modern web browser, such as Google Chrome or Microsoft Edge. The HTML plots have a toolbox that contains wheel/box zoom in/out, pan, freehand draw, save, and reset features that allow the user to interactively navigate the data and customise the display. The user can hover over a signal point to reveal its index value and the signal value. We provide a brief video guide to assist new users in navigating the *Squigualiser* browser view: https://youtu.be/kClYH4KpOjk. *Squigualiser* plots conform to several plot conventions to maintain reproducibility and consistency (Section 9). Similar to the IGV, users can load an annotation file in BED format to assist in navigating the browser view. *Squigualiser* supports the more common 3-column BED format as well as the extended 12-column format. Hence, the user can visualise annotations in different colours, different tracks and with (or without) feature labels (Section 10).

3 *Squigualiser reform and realign* subtools

This section explains the format of the move table generated by the ONT basecallers (Section 3.1), how the move table is projected to our Signal alignment String (ss) format (Section 3.2), and how a preliminary signal-to-reference alignment is obtained using the move table information (Section 3.3). The move table is a signal-to-read alignment format introduced by Oxford Nanopore Technologies (ONT), lacking public documentation. We've compiled this documentation based on available information, recognising that ONT may change the format in the future. In order to support move table based alignments in *Squigualiser*, *reform* subtool converts move table to ss tag. SS tag is documented in **Supplementary Note 1**. *Squigualiser's realign* subtool can calculate a signal-to-reference alignment in conjunction with the read-to-reference alignment information.

3.1 Move table

ONT basecallers output move arrays in SAM/BAM format. The important fields are listed below.

1. Primary field - `read_id`
2. Primary field - basecalled fastq sequence length
3. Primary field - basecalled fastq sequence
4. Auxiliary tag 'ns' - raw signal length
5. Auxiliary tag 'ts' - raw signal trim offset

6. Auxiliary tag 'mv' - move table

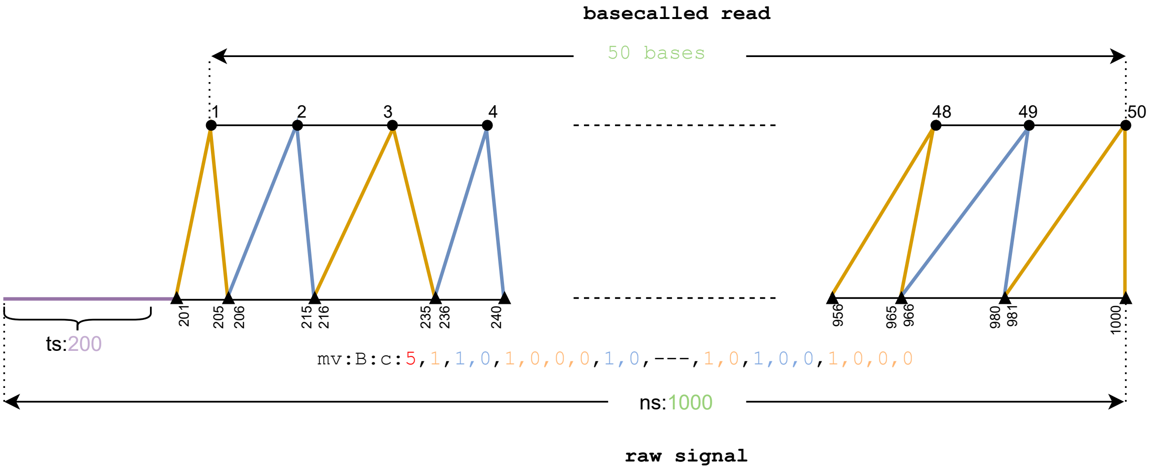

Figure 1: Move table example for signal-to-read alignment

An example move table looks like the following where 'mv' is the tag name and 'B:c:' denotes an array of 'int8_t' elements.

mv:B:c:$5, 1, 1, 0, 1, 0, 0, 0, 1, 0, 1, 0, 1, 0, 0, 0, 1, 0, 1, 0, 1, 1, 0, 1, 0, 1, 0, 1, 1, 1, 1, \ldots$

The downsampling factor used in the neural network (stride) is always the first integer. In the above case it is 5. The rest is the actual move array. The number of ones (1) in the move array equals the fastq sequence length. According to the above example the first move corresponds with 1 x stride signal points. The second move corresponds with 2 x stride signal points. The third with 4 x stride, the fourth with 2 x stride and so on (Figure 1).

The basecalling models have different stride values. The stride values extracted from the move tables generated by Guppy 6.5.7 are listed in Table 1.

Table 1: Strides values of some of the guppy basecaller (v6.5.7) models

Model	Stride
dna_r10.4.1_e8.2_400bps_fast.cfg	5
dna_r10.4.1_e8.2_400bps_hac.cfg	5
dna_r10.4.1_e8.2_400bps_sup.cfg	5
dna_r9.4.1_450bps_fast_prom.cfg	5
dna_r9.4.1_450bps_hac_prom.cfg	5
dna_r9.4.1_450bps_sup_prom.cfg	5
rna_r9.4.1_70bps_hac_prom.cfg	10
rna_r9.4.1_70bps_fast_prom.cfg	12

3.2 *Squigualiser reform* subtool

The move table format is not general format. A move that is not a multiple of the stride and indels cannot be stored in the move table format. Moreover, it can only encode signal-to-read alignments and cannot encode signal-to-reference alignments. A generalised format called ss tag that we introduced is documented in **Supplementary Note 1**. For example, this move string mv:B:c:5:1,1,0,1,0,0,0,0,1,0,1,0,1,0 can be stored in the ss tag format as ss:Z:5,10,25,10,10,10. Furthermore, complex alignments can be stored in the ss tag format. For example, ss:Z:7,2D3,4I,5 should be interpreted as 7 samples match, 2 bases deletion, 3 samples match, 4 samples insertion, followed by a 5 samples match. Such alignments are not directly possible to be encoded in the current version of the move table format.

Therefore, input alignments should be in the ss tag format to generate plots using *Squigualiser*, i.e. move table must be converted to the ss tag format. This is done using *Squigualiser reform* subtool. The two

important parameters *reform* takes are *kmer_length* (or k) and *sig_move_offset* (or m). This determines the best base colour adjustment to the signal events (**Supplementary Note 3**). The user can provide the --*profile* parameter to use predetermined values *kmer_length* and *sig_move_offset* (Table 2).

Table 2: Predetermined *kmer_length* and *sig_move_offset* values for the guppy basecaller (v6.5.7) models

DNA/RNA	Profile Name	Kmer Length	Sig Move Offset
DNA	guppy_dna_r9.4.1_450bps_fast	3	2
DNA	guppy_dna_r9.4.1_450bps_fast_prom	3	2
DNA	guppy_dna_r9.4.1_450bps_hac	3	2
DNA	guppy_dna_r9.4.1_450bps_hac_prom	3	2
DNA	guppy_dna_r9.4.1_450bps_sup	4	3
DNA	guppy_dna_r9.4.1_450bps_sup_prom	4	3
DNA	guppy_dna_r10.4.1_e8.2_400bps_fast	2	1
DNA	guppy_dna_r10.4.1_e8.2_400bps_fast_prom	2	1
DNA	guppy_dna_r10.4.1_e8.2_400bps_hac	2	1
DNA	guppy_dna_r10.4.1_e8.2_400bps_hac_prom	2	1
DNA	guppy_dna_r10.4.1_e8.2_400bps_sup	2	1
RNA	guppy_rna_r9.4.1_70bps_fast	1	0
RNA	guppy_rna_r9.4.1_70bps_fast_prom	1	0
RNA	guppy_rna_r9.4.1_70bps_hac	1	0
RNA	guppy_rna_r9.4.1_70bps_hac_prom	1	0

3.3 *Squigualiser realign* subtool

As discussed before, *Squigualiser reform* converts the basecaller move table to ss format. Subsequently, employing the read-to-reference alignment (CIGAR string) enables the derivation of a signal-to-reference alignment. This is implemented in *Squigualiser realign* subtool as explained in Figure 2 and in Algorithm 1. *Squigualiser realign* subtool produces the signal-to-reference alignment in ss tag format

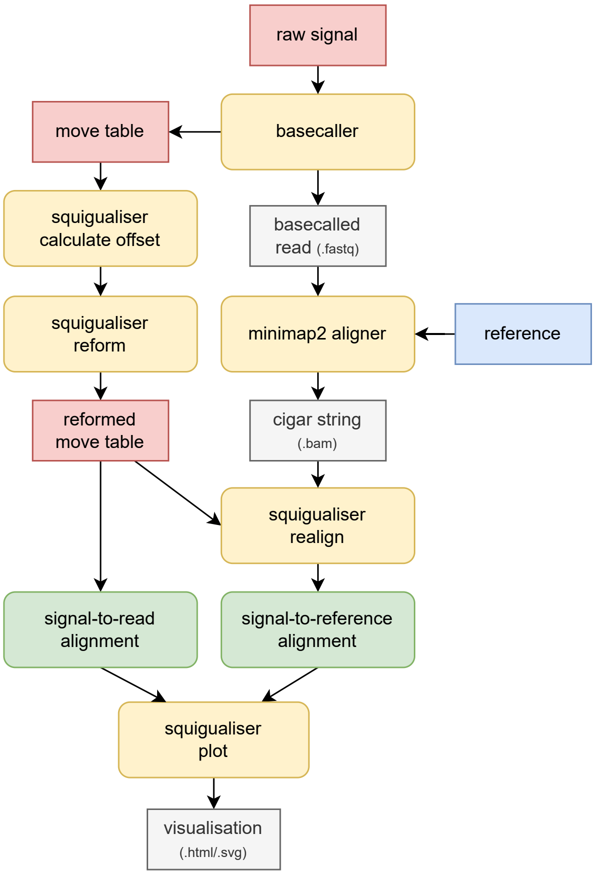

Figure 2: *Squigualiser reform* and *realign* subtools are used for signal-to-read alignment and signal-to-reference alignment respectively.

Algorithm 1: *Squigualiser realign* subtool

Data: CIGAR string (read-to-reference alignment), reformed array (signal-to-read alignment)
Result: reformed array (signal-to-reference alignment)

```
for each position in CIGAR string and reformed array do
    // Process soft-clipped bases
    if current position corresponds to soft-clipped bases then
        Clip corresponding moves from the reformed array;
    // Process insertions
    if current position corresponds to an insertion ('I' in CIGAR string) then
        Add an 'I' operator to the reformed array;
    // Process deletions
    if current position corresponds to a deletion ('D' in CIGAR string) then
        Add a 'D' operator to the reformed array;
    // Handle other CIGAR operations accordingly
    // Handle DNA/RNA accordingly
```

The subtools *reform* and *realign* are used for pre-processing the basecaller's move table to be compatible with plotting subtools in *Squigualiser*. *Reform* takes an unaligned SAM/BAM file containing the move table and generates signal-to-read alignment in PAF format (containing ss tag). *Realign* takes the PAF output from reform along with read alignments in SAM format (e.g., Minimap2 alignments to the reference containing CIGAR string) to output the signal-to-reference alignment in SAM format. The following example commands demonstrate how the basecaller's move table is pre-processed:

```
# transform a signal-to-read alignment from basecaller move table to ss format
squigualiser reform -c --bam movetable.bam -o reform.paf

# create a signal-to-reference alignment using basecaller alignment
squigualiser realign --bam minimap2.bam --paf reform.paf -o realign.bam
```

Plotting alignments from *f5c, Squigulator, Nanopolish* signal projection or *Sigfish* does not require any pre-processing, as we have modified these software to directly output in SAM format containing ss tag. The command-line options along with example commands for these tools to generate such output are described in the *Squigualiser* readme at https://hiruna72.github.io/squigualiser/.

4 *Squigualiser calculate_offsets* subtool

The *calculate_offsets* subtool can be used to perform k-mer-to-base shift correction for more intuitive visualisation (discussed in detail in Supplementary Note 3). This subtool can be invoked either on a PAF file containing the ss tag (e.g., output from reform) or a k-mer model (e.g., Nanopolish/f5c k-mer model). k-mer-to-base shift correction can be performed using the following example command:

```
# calculate the most significant base index using a PAF file containing ss tag
squigualiser calculate_offsets -f reads.fastq -s reads.blow5 -a reform.paf

# calculate the most significant base index using a k-mer-model
squigualiser calculate_offsets --use_model kmer.model
```

For the user's convenience, the k-mer-to-base shift correction values for known k-mer models and basecaller models are provided as profiles (–profile) in *Squigualiser plot* and *plot_pileup* subtools (see Supplementary Note 2, Table 2 and Supplementary Note 3, Table 2).

5 *Squigualiser plot, plot_pileup and plot_tracks* subtools

Squigualiser has the subtools *plot, plot_pileup* and *plot_tracks* for plotting the data. The *plot* subtool is used to generate a plot for a single signal-to-read alignment or a single signal-to-reference alignment. For a signal-to-read plot, the inputs must be a PAF file containing ss tags (reform, f5c resquiggle or Squigulator PAF output), the raw signal file in BLOW5 format[2] and the basecalled read in FASTQ format (Fig3b). For a signal-to-reference plot, the required inputs are a BLOW5 file, reference genome/transcriptome in FASTA format and signal-to-reference alignment in BAM/PAF format containing ss tags (Fig3c). This BAM/PAF file can be the output of realign, f5c eventalign, etc (Fig3c). The *plot_pileup* subtool is used to generate signal-to-reference alignment plots in 'pileup' format, where individual reads are stacked vertically. The input for

plot_pileup is the same as for the *plot* subtool above for signal-to-reference alignments (Fig3c). To visualise multiple pileups in a single HTML file (e.g., pileups from different samples or alternative alignment methods), the *plot_tracks* subtool can be used. *plot_tracks* accepts a text file with each *plot_pileup* command in a separate line. The basic structure of the plotting commands is listed below.

```
# plot a signal-to-read alignment
squigualiser plot -f reads.fastq -s reads.blow5 -a reform.paf -o output_html_dir

# plot a signal-to-reference alignment
squigualiser plot -f genome.fasta -s reads.blow5 -a realign.bam --region chrX:N-M -o
    output_html_dir

# plot a signal-to-reference pileup
squigualiser plot_pileup -f genome.fasta -s reads.blow5 -a realign.bam --region chrX:N-M
    -o output_html_dir

# plot multiple signal-to-reference pileups
squigualiser plot_tracks -f commands.txt -o output_dir
```

The user is provided with multiple data plotting and signal adjustment options in *plot, plot_pileup* and *plot_tracks* subtools. For example, the user can change the plot dimensions, initial x-range, hide base colours/signal samples etc. Further, the user can opt for pA conversion and several signal normalisation methods including Z-score scaling, Median-Median-Absolute-Difference scaling (med-MAD)[3], and scaling to the k-mer model. In signal pileup mode the user can limit the number of signals to plot as well as the maximum number of signal points per signal. This will enhance user experience as the responsiveness of the plots can vary depending on the compute resources (GPU, Display resolution and size).

6 Strategies behind fast region retrieval

If the user wants to plot a specific reference region, then the sequence file (in FASTA format) must be indexed (faidx) and the signal-to-sequence alignment (in SAM/PAF formats) file must be sorted and indexed. This is important as it is necessary to fetch only the necessary reads spanning across a given reference region. SAM format is preferred as it is well supported through *samtools* and its binary format (BAM) enables efficient region-based access. If PAF is used, the user has to sort it manually by providing the correct column indices for DNA/RNA, convert it to *bgzip* and index using *tabix*. The raw signal which is the largest portion of the input data must be in BLOW5 format indexed using *slow5tools*[4], to enable efficient built-in random access functionality. The possibility to fetch only the necessary raw signals (random access) is vital to plot signal pileups spanning across a given reference region. Python package pyslow5 is used to handle BLOW5 format files. These input formats enable *Squigualiser* to seamlessly generate signal pileup plots for a given reference region regardless of the size of the dataset. The retrieval of aligned records for the user specified region (ref_name:ref_start-ref_end) from SAM/PAF file, followed by fetching the auxiliary tags (e.g., ss tag) for each alignment record, and then fetching the relevant raw signal from the SLOW5 file can be done as follows.

```
# fetching a region from SAM
alignment_record = sam_file.fetch(contig=ref_name, start=ref_start, stop=ref_end)
# fetching a region from PAF
alignment_record = paf_file.fetch(ref_name, ref_start, ref_end)

# fetching the ss auxiliary tag
signal_to_reference_alignment = alignment_record.get_auxiliary_tag(ss)
# fetching the raw signal from SLOW5
signal = slow5_file.get_read(alignment_record.read_id)
```

The fetched alignment_record is not guaranteed to exactly start and finish at the user-specified region. Instead, it can start before the user-specified start position and end before the user-specified end position. Hence, *Squigualiser* further refines the alignment_record and the signal_to_reference_alignment to get only the exact interval before generating plots.

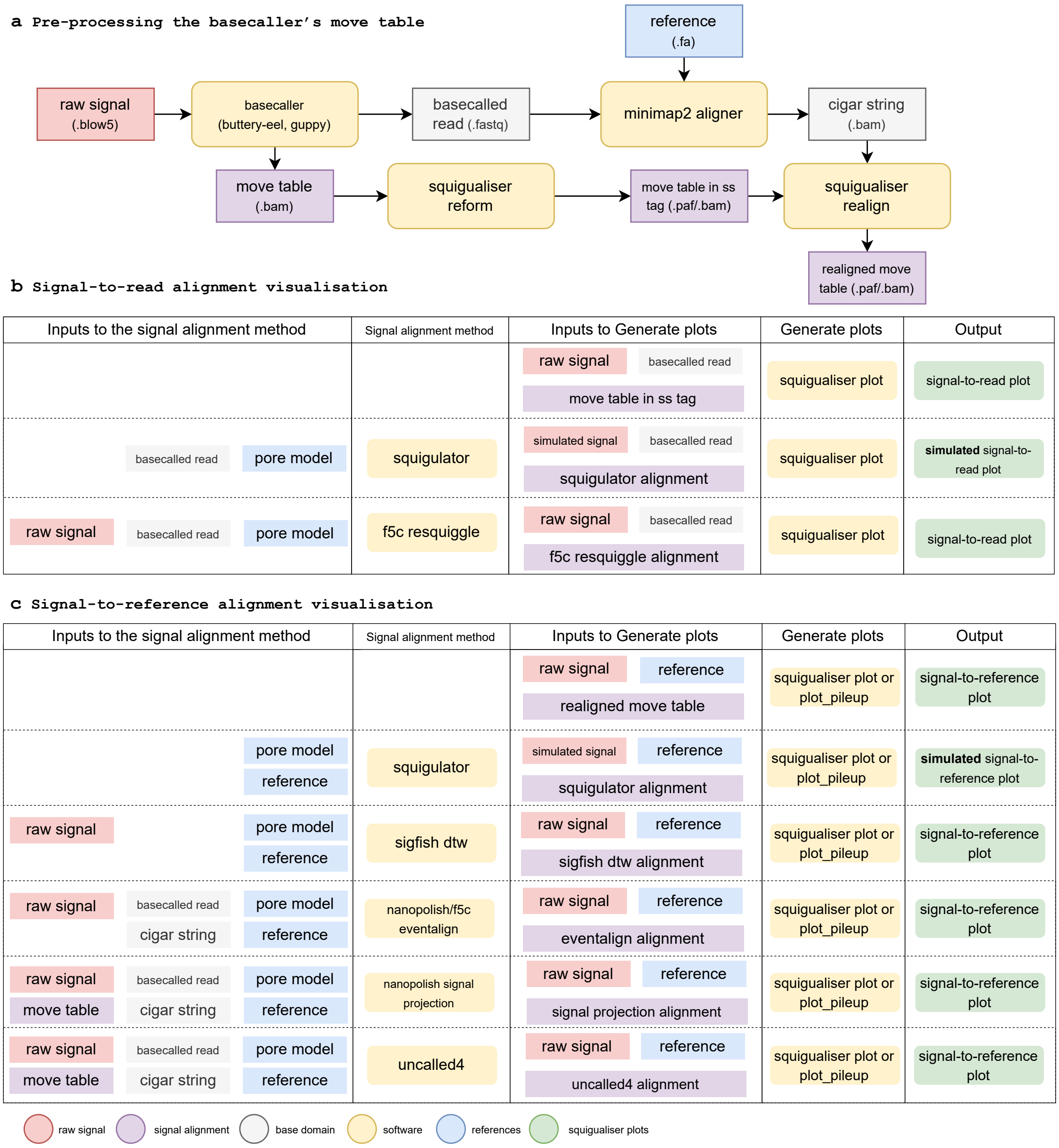

Figure 3: **Overview of *Squigualiser* visualisation framework.** Schematic diagram summarises data pre-processing steps and alternative workflow paths to signal alignment visualisation with *Squigualiser*. Diagram is colour coded as follows: signal data domain (red); basecalled sequence data domain (white); ONT move table (purple); constants (blue); software programs (yellow). (a) The *calculate_offset* and reform subtools within *Squigualiser* may be used to convert the move table generated by ONT basecallers (*Guppy*/*Dorado*) into signal-to-read alignment format with ss tags, which can be visualised directly, or can be translated to signal-to-reference alignments using the realign subtool (b). (c) Alternatively, the user may perform signal-to-reference alignment with a variety of external software, including *F5c*, *Nanopolish*, *Uncalled4* or *Sigfish*. *Squigualiser plot* (single read) or *plot_pileup* (multiple reads in pileup format) are used to generate an interactive browser view (HTML format) capturing a specified set of reads and/or reference region, which can be displayed and navigated in a standard web browser (an example is provided in Supplementary_File_1.html). Our recent tool for signal data simulation, *Squigulator*, may be used to generate simulated signal-to-read and signal-to-reference alignments, which may be plotted as 'expected data' tracks by *Squigualiser*.

7 Signal storage formats

Since FAST5 and POD5 file formats do not have direct random access capability *Squigualiser* only supports SLOW5/BLOW5 format. The user is advised to use *slow5tools* (https://github.com/hasindu2008/slow5tools) and *blue-crab* (https://github.com/Psy-Fer/blue-crab) respectively to convert FAST5 and POD5 formats to SLOW5/BLOW5.

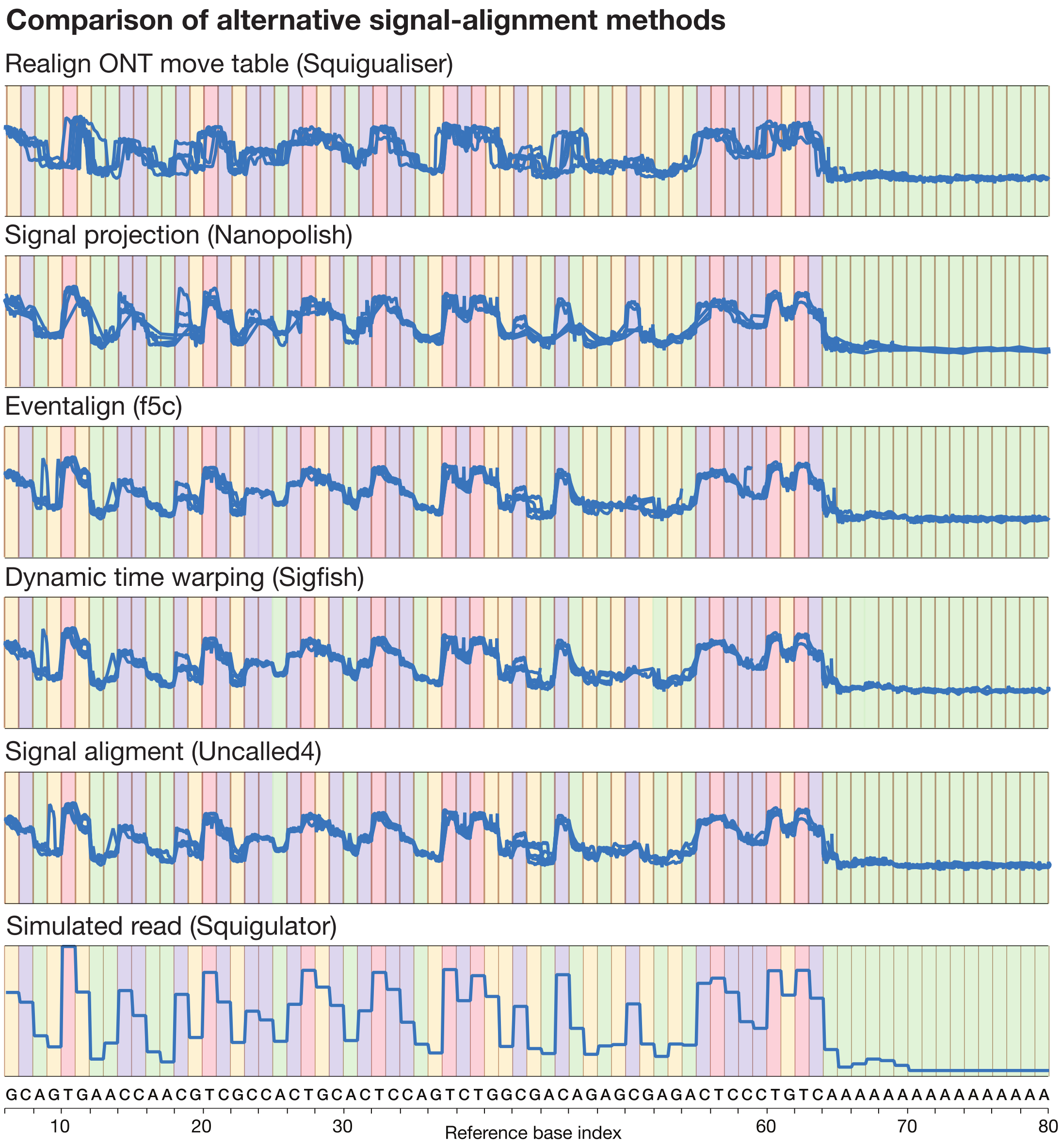

Figure 4: **Using *Squigualiser* to evaluate alternative signal alignment methods.** Exported *Squigualiser* pileup images show a matched set of signal-to-reference alignments generated using four alternative methods: alignments extracted directly from the ONT move table using *Squigualiser* reform and realign subtools; alignments generated using the signal projection method in the *Nanopolish* toolkit; alignments generated using the event alignment method in the *F5c* toolkit; alignments generated using *Sigfish* dynamic time warping for signal mapping; alignments generated using *Uncalled4* signal alignment method. The bottom track shows simulated signal data from *Squigulator*, as a guide.

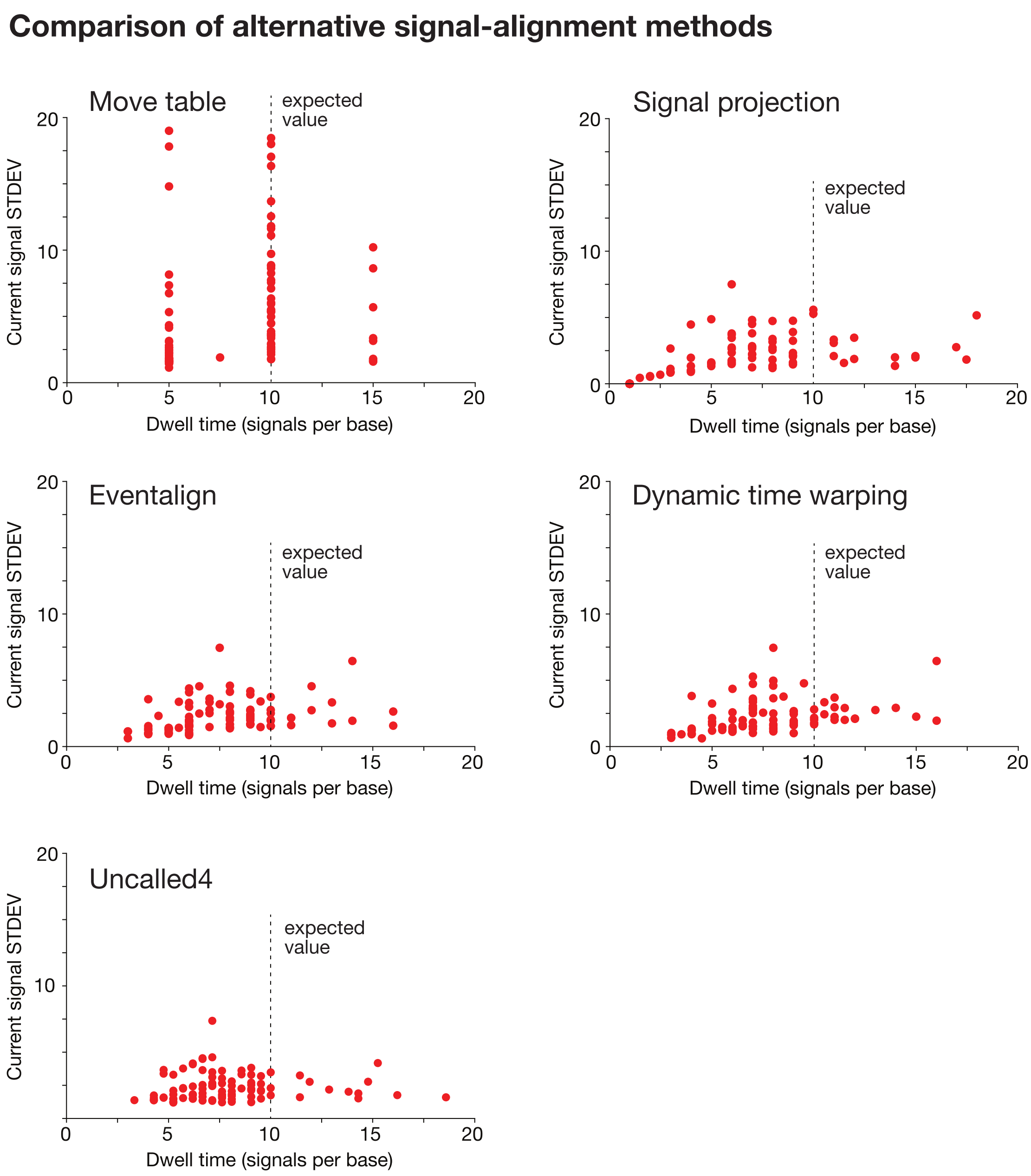

Figure 5: **Using *Squigualiser* to evaluate alternative signal alignment methods.** The alternative methods above can be statistically evaluated on the precision/uniformity of signal alignments. Dots plots here show standard deviations for all signal data values aligned to each individual reference base (each base is a single red dot) on the vertical axis and the number of signal values per base per read on the horizontal axis. *F5c* eventalign, DTW and *Uncalled4* show the lowest variation across the two domains, and are generally our preferred methods for signal-to-reference alignment.

8 Visualilsing different alignments

The user may opt to use various external signal alignment software, including *Squigualiser realign* for signal-to-read alignment, or *F5c* eventalign, *Nanopolish* signal projection, *Sigfish* dynamic time warping, or *Uncalled4* for signal-to-reference alignment (Fig 4 & 5). Each approach has different merits and the user is free to choose their preferred method, depending on the context. Moreover, in supporting different methods, *Squigualiser* provides a framework to compare and evaluate them. For example, when assessed by the precision and uniformity of signal alignments across multiple independent reads, *F5c* eventalign, DTW and *Uncalled4* achieve superior

performance and is generally our preferred approach (Fig 4 & 5). Although this analysis shows the *realign* method to be the least precise, this method has the advantage of being non-reliant on a k-mer pore model or external alignment software.

9 Plot conventions

For the best user experience *Squigualiser* conforms to the plot conventions that are described in Fig. 6.

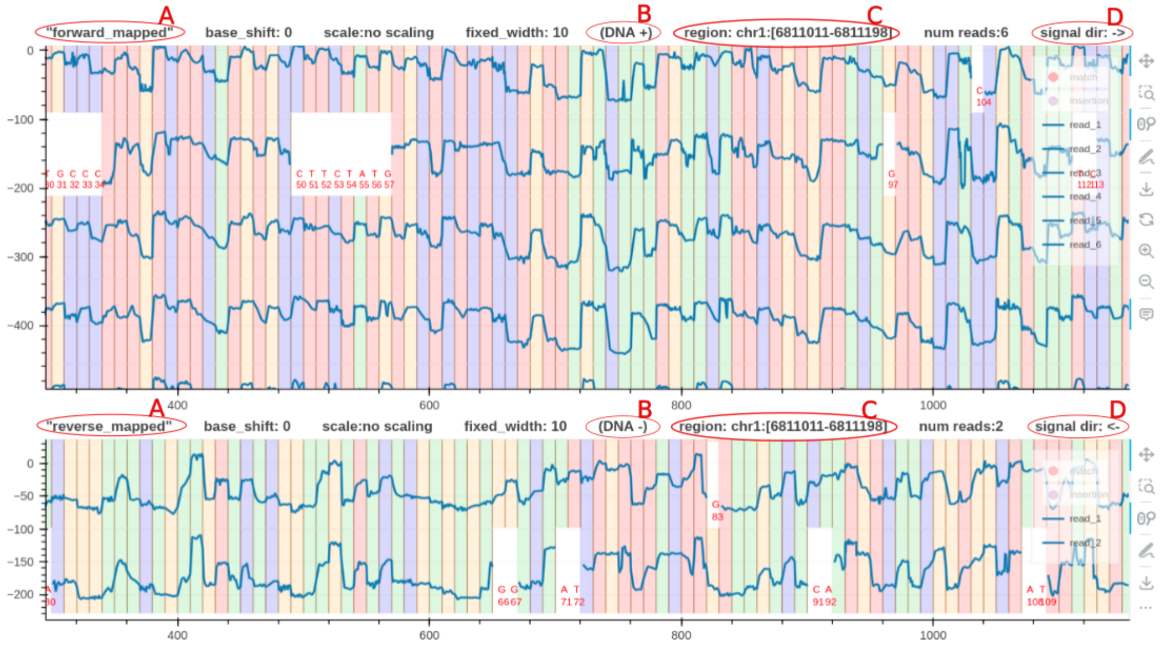

Figure 6: Plot conventions of squigualiser
A is a descriptive tag name to identify the plot.
B indicates whether the positive or negative strand was used as the reference to align the signals. For RNA this will be RNA 3'->5'. *Squigualiser* only supports RNA reads mapped to the transcriptome.
C always indicates the region using the positive strand coordinates, regardless of the forward and reverse mapped plots.
D indicates the true sequencing direction of the signals.

10 Plot annotations

Squigualiser plot and *plot_pileup* can take a BED file that has annotation information. The BED format can store color values and BED line names in addition to the genomic positions. The BED file used in generating the signal pileup shown in Fig. 7 had three BED lines (*POS1, POS2, and POS3*) and hence, the plot has three BED lines.

```
squigualiser plot_pileup [OPTIONS] --bed annotation.bed --print_bed_labels -f reference.
    fa -s reads.blow5 -a eventalign.bam --region region
```

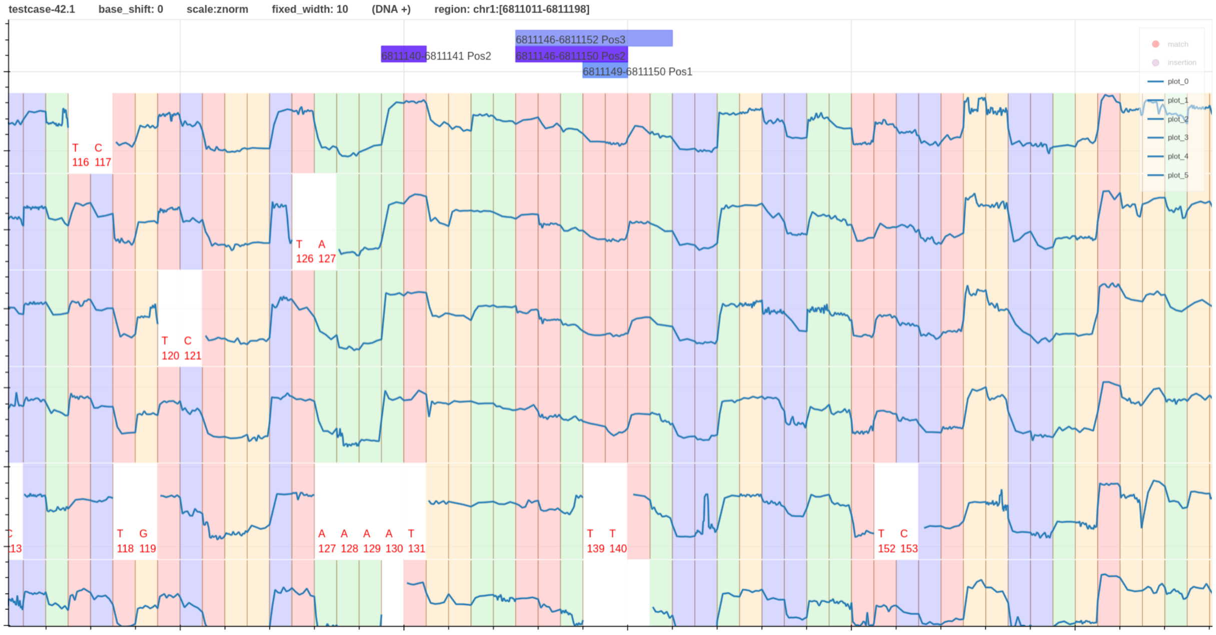

Figure 7: DNA signal pileup with three layers of annotation loaded using a single BED file.

11 Data & Code availability

The HG002 dataset sequenced on an ONT R10.4.1 PromethION flowcell used for the figures is publicly available on the NCBI Sequence Read Archive (SRR23215366). The synthetic unmodified and modified RNA datasets used in Supplementary Note 5, are also publicly available on NCBI SRA (SRR22888949, SRR22888950).

Squigualiser and all modified software associated with the manuscript is free and open source under an MIT licence: *Squigualiser*: https://github.com/hiruna72/squigualiser

F5c with ss tag support: https://github.com/hasindu2008/f5c/releases/tag/v1.4

Squigulator with ss tag support: https://github.com/hasindu2008/squigulator/releases/tag/v0.2.2

Sigfish with ss tag support: https://github.com/hasindu2008/sigfish/releases/tag/v0.2.0-alpha

Nanopolish signal projection with ss tag support:

https://github.com/hiruna72/nanopolish/tree/215592e0913418c2b20a86a67483cc1c6f557ca8

All the pipeline scripts, curated datasets, and generated plots can be found in the *Squigualiser* repository: https://github.com/hiruna72/squigualiser/tree/main/test/data/raw/pipelines

The user should be able to reproduce all our results by amending the scripts as explained in the associated documentation: https://hiruna72.github.io/squigualiser/docs/pipeline_basic

References

[1] James M Ferguson et al. "InterARTIC: an interactive web application for whole-genome nanopore sequencing analysis of SARS-CoV-2 and other viruses". In: *Bioinformatics* 38.5 (2022), pp. 1443–1446.

[2] Hasindu Gamaarachchi et al. "Fast nanopore sequencing data analysis with SLOW5". In: *Nature biotechnology* 40.7 (2022), pp. 1026–1029.

[3] James M Ferguson and Martin A Smith. "SquiggleKit: a toolkit for manipulating nanopore signal data". In: *Bioinformatics* 35.24 (2019), pp. 5372–5373.

[4] Hiruna Samarakoon et al. "Flexible and efficient handling of nanopore sequencing signal data with slow5tools". In: *Genome Biology* 24.1 (2023), p. 69.
