# Supplementary material for: Interactive visualization of nanopore sequencing signal data with Squigualiser
Source: Bioinformatics. 2024 Aug 13;40(8):btae501. doi: 10.1093/bioinformatics/btae501 (PMC11335371; doi:10.1093/bioinformatics/btae501)
Supplement: btae501_Supplementary_Materials [file btae501_supplementary_materials.zip › SupplementaryMaterials/Supplementary Note 3.pdf]

Supplementary Note 3: Strategies behind signal alignment visualisation enhancements

Hiruna Samarakoon, Kisaru Liyanage, James M. Ferguson, Sri Parameswaran, Hasindu Gamaarachchi, Ira W. Deveson

July 1, 2024

Note: Read this document sequentially. Skipping sections without prior context is not recommended, as the content is not reiterated.

This document first explains the concept of k-mer model and the ***Most Significant Base*** **(MSB)** in Section 1. Then, Section 2 presents the technical details about calculating the MSB. Section 3 demonstrates how the correct application of the MSB concept can be further validated using an example involving heterozygous A/T SNV.

1 K-mer model & MSB

Different nanopore chemistries have different pore specifications, thus, different k-mer lengths, current levels and standard deviations. This information is stored in a file called k-mer model/table (also known as pore model/table). Many signal alignment methods including *Nanopolish/f5c eventalign, Nanopolish signal projection, Squigulator simulation*, and *Sigfish dtw* use k-mer models. At a given moment, all the bases of the k-mer inside the nanopore influence the current/voltage level. However, not all the bases equally contribute to this phenomenon (Fig. 1). Our aim is to visualise the MSB (that mostly influences the current level) and the relevant signal event together.

Depending on the method used to generate the k-mer model, the central base of the k-mer may or may not be the MSB (Table 1). Given a pore-model, we can find the position of the MSB (that influences the k-mer current level the most) in the k-mer, by analysing the degree to which each base in the k-mer can discriminate A, C, G, and T/U. For instance, for a 6-mer model, initially (iteration round 0), we assume the first base of the k-mer is the MSB (Fig. 2 first plot titled *base shift:0*). Then we create a 1-mer table by ignoring the rest of the bases in the k-mer. There can only exist 4 different 1-mers (A, C, G, T/U), i.e., the current levels for AXXXXX and AYYYYY both now get mapped to 1-mer A. That is a many-to-one mapping (1024 current level mappings to 1-mer) per each 1-mer. There will be 4 current level distributions (each distribution

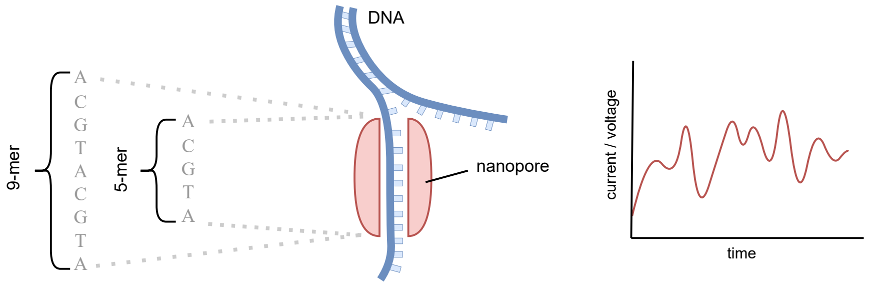

Figure 1: Depending on the size of the nanopore the k-mer inside the pore varies in length. In addition, depending on how the k-mer-model was generated, the MSB may or may not be the base at the center of the k-mer.

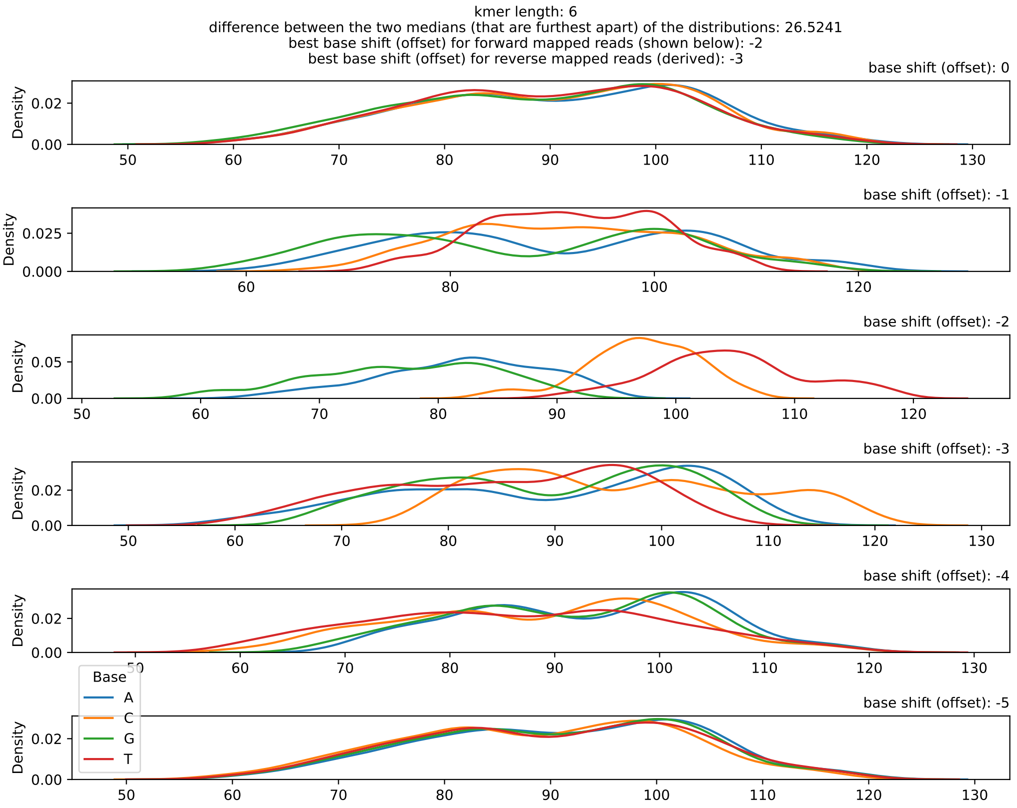

Figure 2: DNA R9.4.1 6-mer model

containing 1024 current levels) per base A, C, G, and T/U (Fig. 2 first plot titled *base shift (offset) :0*). The narrower the individual distributions are and the more the 4 distributions are separated from each other; the stronger our assumption becomes. In the next iteration (round 1), we assume the second base of the k-mer is the MSB and repeat the experiment (Fig. 2 second plot titled *base shift (offset):-1*). Likewise, we do k iterations (Fig. 2). Then we choose the iteration where the 4 distributions had the best level of separation (third plot titled *base shift (offset):-2* in Fig. 2 that relates to iteration 2).

Table 1: K-mer Model specifications (*Nanopolish/f5c* models)

**Chemistry**	**k-mer size**	**MSB index (0-based)**	**Figure No.**
DNA R9.4.1	6-mer	2	2
RNA R9.4.1	5-mer	1	3
DNA R10.4.1	9-mer	6	4

In DNA models, looking at the current level distributions for each nucleotide of the MSB index, we observe that the highest median current is for the nucleotide ‘T’ followed by the second highest median current for the nucleotide ‘C’. The median currents for the nucleotide ‘A’ and ‘G’ are the lowest and they are close to each other.

To generate similar density plots for a new k-mer model, the following command can be used (more details in section 2).

```
squigualiser calculate_offsets --use_model --model ${MODEL_PATH} -o ${OUTPUT_PDF_PATH}
```

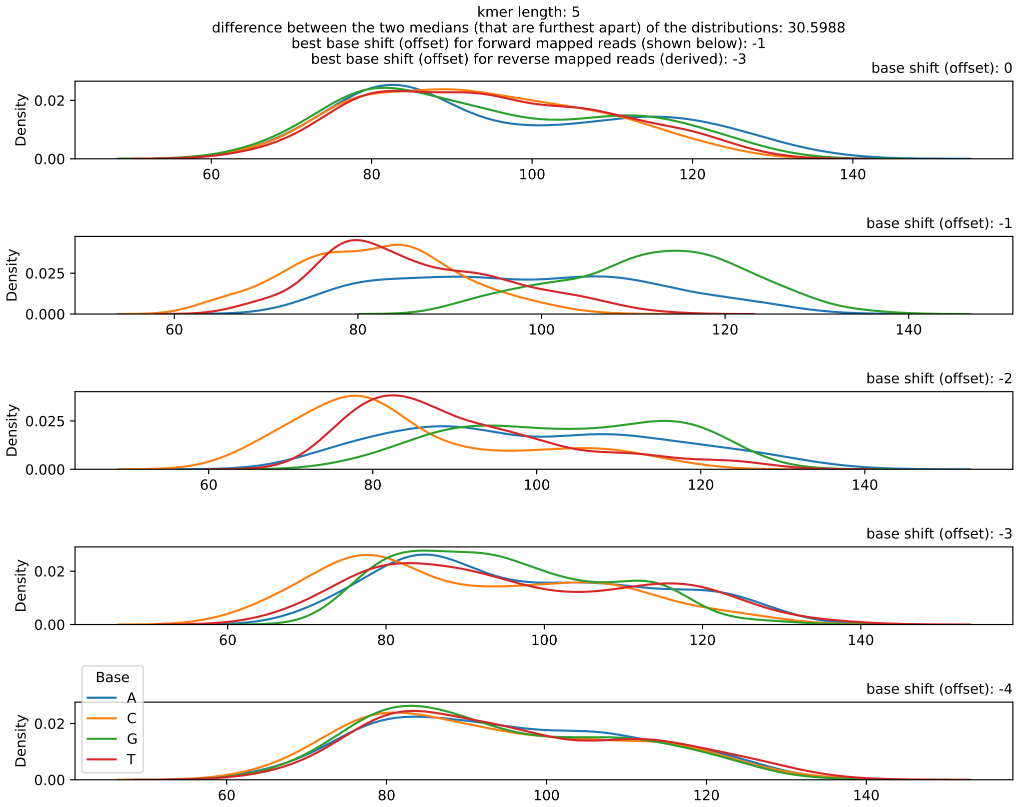

Figure 3: RNA R9.4.1 5-mer model

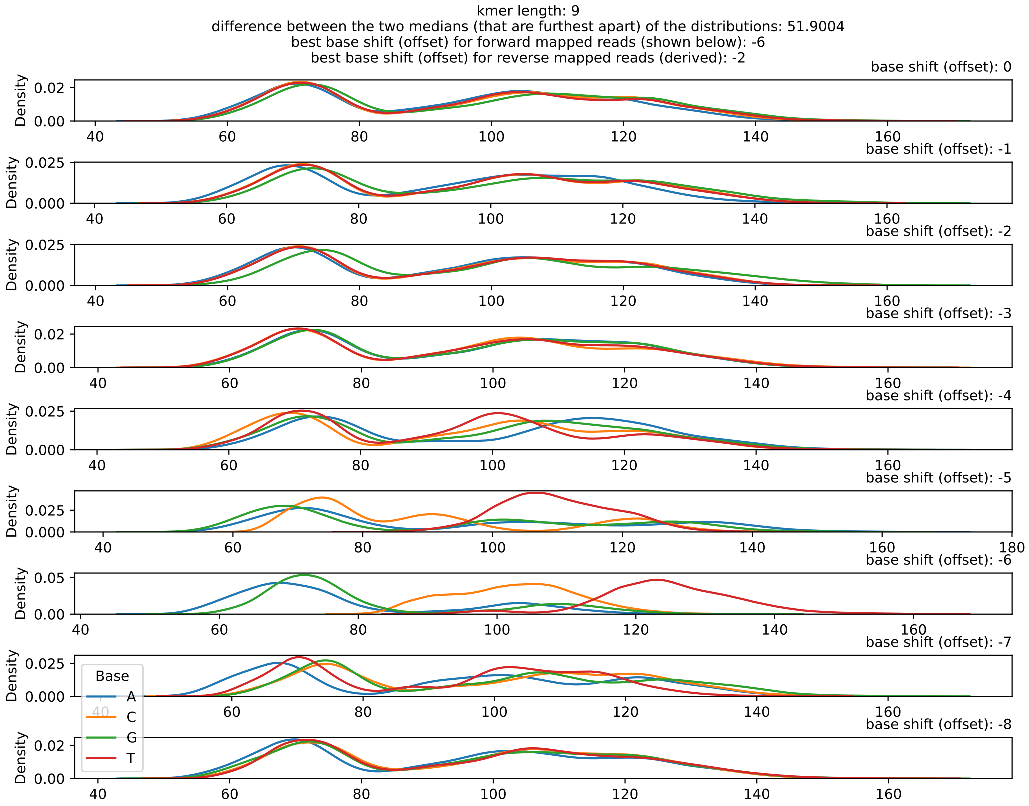

Figure 4: DNA R10.4.1 9-mer model

2 Calculating the MSB index

As the reader is now familiar with the concept of the MSB, we will now delve into details of the *Squigualiser calculate_offsets* subtool. *calculate_offsets* can calculate the signal-to-MSB alignment and has two modes. As mentioned in the main text, k-mer-to-base shift correction is necessary for both signal-to-kmer alignment (explained in *Mode 1*) and signal-to-base alignment (explained in *Mode 2*, **Supplementary Note 2**).

2.1 Mode 1

Squigualiser calcualte_offsets when run in *Mode 1* setting will calculate the MSB of a k-mer model. Subsequently, any signal-to-k-mer alignment that used the k-mer model should be transformed to signal-to-MSB alignment. This transformation is done inside *Squigualiser*. *Nanopolish/f5c* and *Squigulator* default k-mer models are listed as profiles in Table 2. The user can provide the profile name when using *Squigualiser plot* or *plot_pileup* subtools. An example is given below.

```
squigualiser plot_pileup [OPTIONS] --profile profile_name -f genome.fasta -s reads.blow5
    -a eventalign.bam -o output_dir --region region
```

If the signal alignment method used a custom k-mer model then the user is advised to run *calcualte_offsets* to find the most signficant base index of the k-mers in the model as follows.

```
squigualiser calculate_offsets --use_model --model ${MODEL_PATH} -o ${OUTPUT_PDF_PATH}
```

Above command will output *base_shift* values for forward mapped reads and reverse mapped reads. The values can be used in *Squigualiser plot* or *plot_pileup* as follows.

```
squigualiser plot_pileup [OPTIONS] --base_shift forward_base_shift -f genome.fasta -s
    reads.blow5 -a eventalign.bam -o output_dir --region region
```

```
squigualiser plot_pileup [OPTIONS] --plot_reverse --base_shift reverse_base_shift -f
    genome.fasta -s reads.blow5 -a eventalign.bam -o output_dir --region region
```

The derivation of the *base shift* value for reverse mapped reads is illustrated in Fig. 5

Table 2: Model Parameters

k-mer Model Profile	Offset (Forward)	Offset (Reverse)
kmer_model_dna_r9.4.1_450bps_5_mer	-2	-2
kmer_model_dna_r9.4.1_450bps_6_mer	-2	-3
kmer_model_rna_r9.4.1_70bps_5_mer	-3	-1
kmer_model_dna_r10.4.1_e8.2_400bps_9_mer	-6	-2

The pseudo code given below explains the computation done inside *calculate_offsets Mode 1*.

```
find_most_significant_base(kmer_model){

    for base_index in kmer_length{
        for kmer, current_level in kmer_model{
            bin current_level to one of the four bins A,C,G and T
        }

        draw four density distributions, one for each bin
        calculate difference (diff) between highest and lowest medians of the
    distributions
    }

    select the maximum difference (max_diff) among the differences (diff)
    pick the corresponding base index (base_index) as the MSB index

    return  max_diff, base_index
}
```

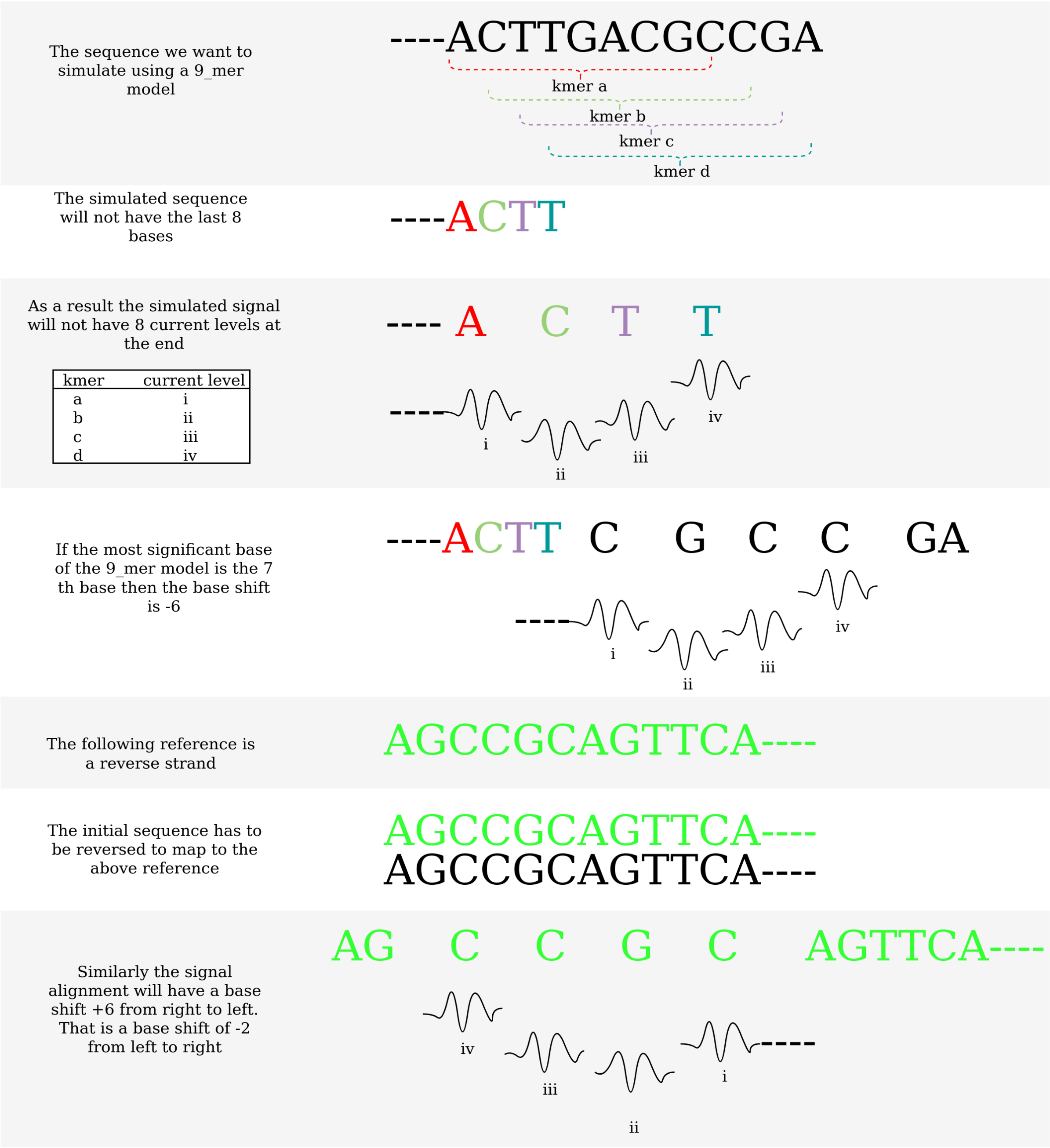

Figure 5: Illustration of deriving correct *base shift* value for reverse mapped sequences

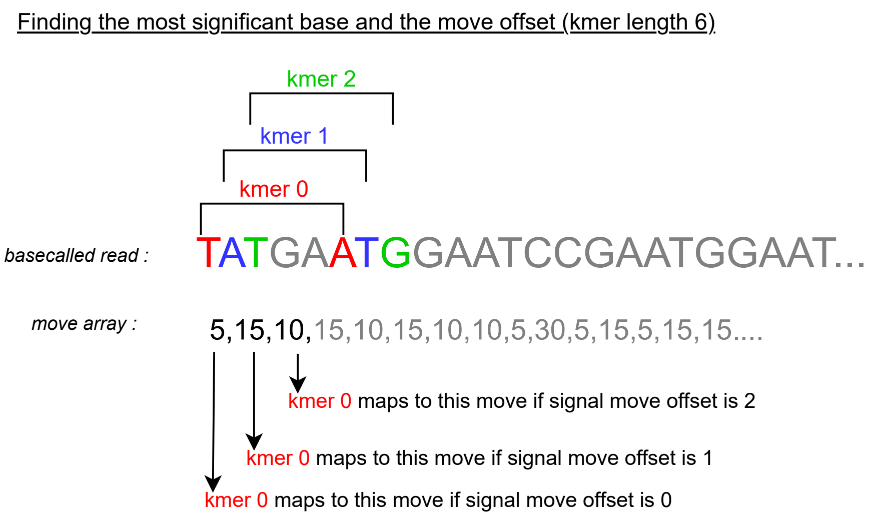

Figure 6: Illustration of how *squigualiser calculate_offsets* Mode 2 finds the best *kmer_length* and *sig_move_offset* to generate the signal-to-MSB alignment.

2.2 Mode 2

As explained above and in **Supplementary Note 2**, even though basecaller's move table is a signal-to-base alignment, depending on the basecalling model it might not be the signal-to-MSB alignment. *Squigualiser calcualte_offsets* when run in *Mode 2* setting will output *kmer_length* and *sig_move_offset* values to be used in *Squigualiser reform* subtool.

```
squigualiser calculate_offsets -p input.paf -s reads.blow5 -f reads.fastq
```

```
squigualiser reform [OPTIONS] -k kmer_length -m sig_move_offset -c --bam movetable.bam -o
     reform.paf
```

The following pseudo code and Fig. 6 should help in understanding how the MSB index is determined in Mode 2.

```
DEFAULT_KMER_LENGTH = 6
best_m = 0
best_max_diff = -infinity
most_significant_base_index = 0
recommended_sig_move_offset = 0
for m in range(0,DEFAULT_KMER_LENGTH):
 # create a kmer model by iterating the fastq sequence, assigning each kmer the current
    value pointed by the move and pass it to Mode 1
    local_max_diff, base_idx = finding_the_most_significant_base(kmer model)
    if best_max_diff < local_max_diff:
        best_max_diff = local_max_diff
        best_m = m
        most_significant_base_offset = base_idx
        if most_significant_base_offset == 0:
            recommended_sig_move_offset = m
# assumption: the recommended_sig_move_offset is the m value where the
    most_significant_base_offset is zero and as we increment m by 1
    most_significant_base_offset also gets incremented by 1)

# confirm that the assumption is valid by checking...
if recommended_sig_move_offset == best_m - most_significant_base_offset
    then recommended_kmer_length = recommended_sig_move_offset + 1
# else increment the DEFAULT_KMER_LENGTH by 1 and further analyse the density plots
    generated.
```

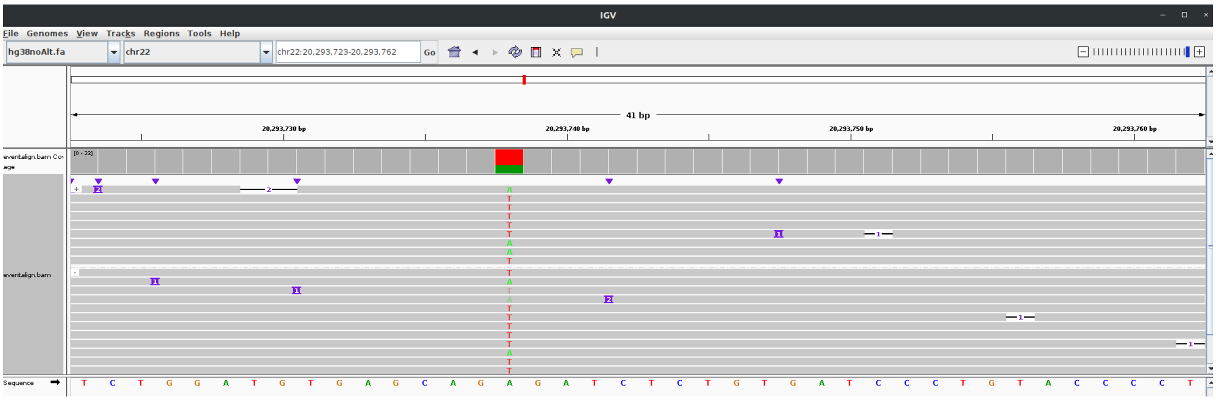

Figure 7: IGV reference-to-read pileup with heterozygous A/T SNV at chr22:20,293,723-20,293,762
.

Mode 2 can optionally take a read id to plot a density plot (similar to Fig. 2, 3, and 4) for the particular signal-to-read alignment as follows,

```
squigualiser calculate_offsets -p input.paf -s reads.blow5 -f reads.fastq -o out.pdf --
    read_id READ_ID
```

2.3 Automatically calculate the MSB

A novice user who is only interested in generating the plots, can skip the MSB index calculation and directly run a plotting tool by passing the argument '–auto' which will attempt to calculate the MSB index on the go.

```
squigualiser plot[OPTIONS] --auto -f reads.fastq -s reads.blow5 -a reform.paf -o
    output_html_dir
squigualiser plot_pileup [OPTIONS] --auto -f genome.fasta -s reads.blow5 -a eventalign.
    bam -o output_dir --region region
```

3 Validating the k-mer-to-base-shift correction using an example

3.1 Preprocessing

In this example let's look at a heterozygous A/T SNV found in the humangenome (hg38). This example only focus on forward-mapped reads. At site chr22:20,293,738 there is A (Read 1,7, and 8) and T (Read 2,3,4,5,6, and 9)(Fig. 7).

Fig.8 shows three signal alignments. The first is the signal-to-reference alignment using *f5c eventalign*. The second and the third are the ideal simulated signals using the reference with and without the SNV respectively. Grey circles mark the SNV we are interested in. As shown in the simulated signals there must be a clear jump up in the signals at A21 when it is a T (third track). However, the *eventalign* algorithm has failed to properly align it the specific position for the reads that have the SNV. It has introduced deletions, and aligned the jump before (at A19) or after (at T24). This phenomenon is not correct but expected as the reads were aligned to the original reference where an A was present, not a T. To rectify this we aligned the reads again to a new reference where the particular A was replaced with a T. Then, we executed *eventalign* with the new read alignment file and the new reference. Subsequently, we selected the signal alignment records from the correct *eventalign* outputs (Read 1,7,8 and 2,3,4,5,6,9 extracted from the original and new outputs respectively). Then we merged the signal alignment records to create a single signal alignment file. It was used to generate a better signal alignment pileup (Fig. 9). This pre-processing workflow is summarised in Fig. 10.

3.2 Statistical evaluation

To evaluate our new k-mer-to-base shift correction strategy, we extracted all signal values aligned to the site of a known heterozygous SNV within the HG002 genome reference sample, repeating this for a set of 250 A-to-T SNVs on chr22 across a range of possible offset values (0 to -8). We observed the highest degree of

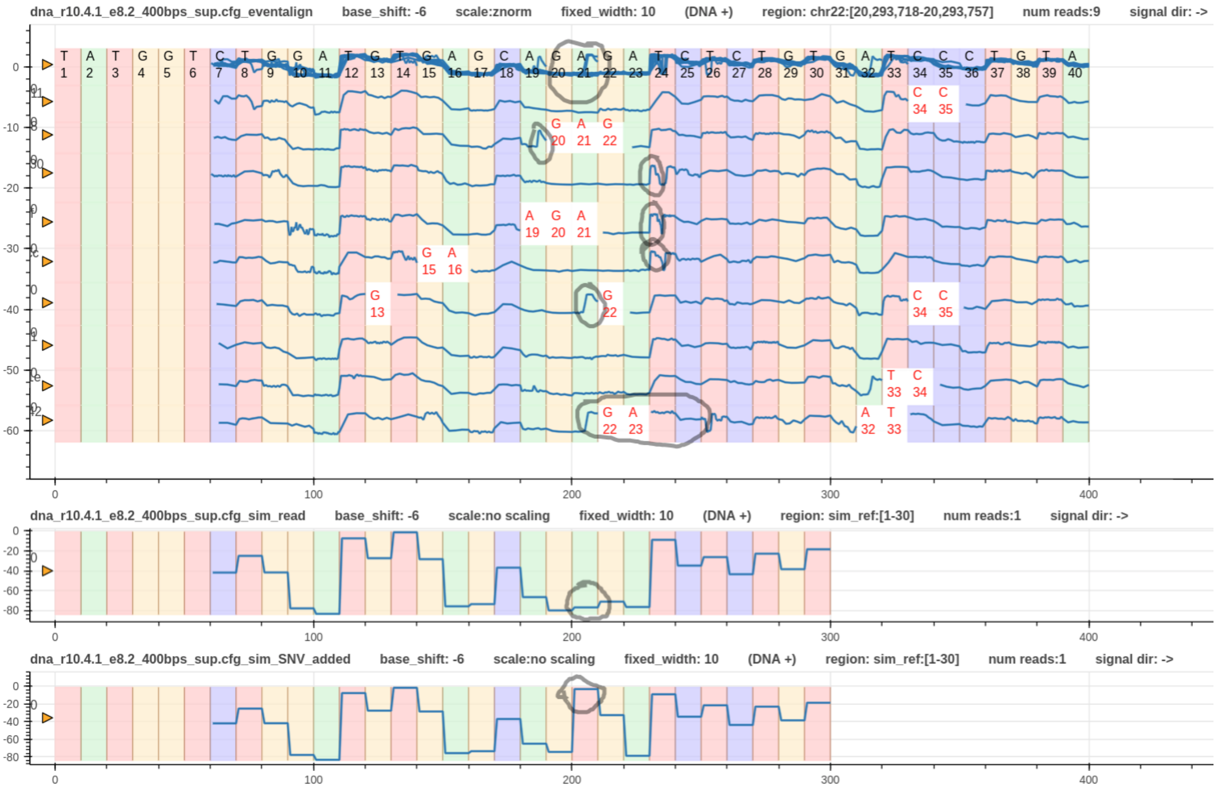

Figure 8: First signal alignment track is the *f5c eventalign*. The second and third tracks show the simulated (expected) ideal signal shape using *squigulator* with and without the SNV, respectively

.

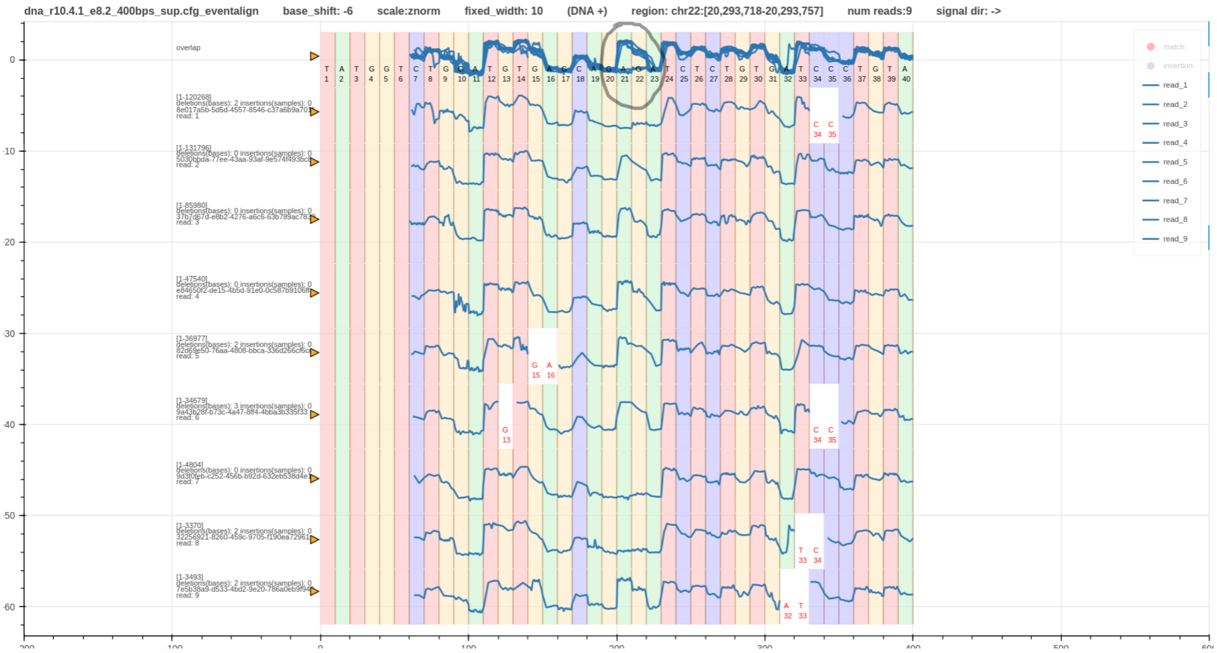

Figure 9: The signal aligment pileup after aligning the signals to the correct reference, i.e., the signals with the SNV were aligned to the reference with the SNV

.

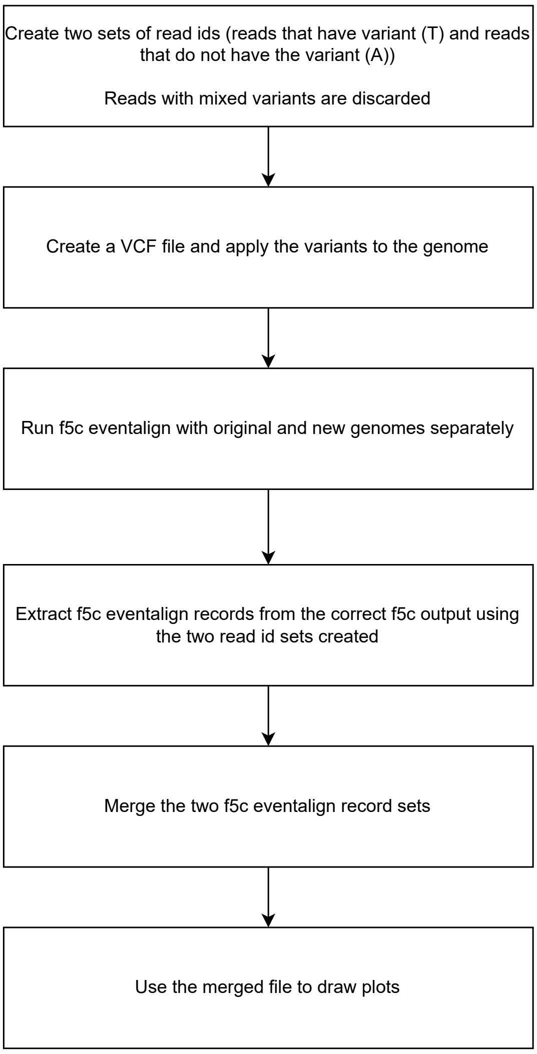

Figure 10: The preprossessing workflow to generate more sensible *eventalign* signal alignment plots
.

Table 3: Comparison of different methods to calculate MSB

	Remora (*Tombo*)	*Uncalled4*	*Squigualiser*
Method to determine most significant base (MSB)	a	b	c
Provide a separate tool (*Squigualiser calculate_offsets* Mode 1) to calculate MSB for a given k-mer model			+
Provide a separate tool (*Squigualiser calculate_offsets* Mode 1) to calculate MSB for a move table (kmer free alignment)			+
Provide a user argument to align the plots to MSB		+	+
Automatically calculate and adjust the plot to MSB for a k-mer-to-signal alignment	+		+
Automatically calculate and adjust the plot to MSB for a move table alignment			+

a - Kruskal-Wallis H-test statistic method (Undocumented).
b - Compute substitution profile for each base in k-mer to find central base.
c - Find position with maximal discrimination between signal density plots for individual bases 'A' 'C' 'G' 'T/U'

bimodality among signal values aligned to heterozygous SNVs when applying a k-mer-to-base shift correction of -6, which was also the optimum offset value calculated by *Squigualiser* for this protocol (Fig 11 & 12). Without any correction (offset = 0), signal values appeared unimodal at heterozygous SNV sites, thereby failing to capture expected current differences between the reference and alternate bases, which are typically visible at a nearby position (Fig 11 & 12). This analysis demonstrates how our k-mer-to-base shift correction approach leads to improved resolution in signal-alignment visualisation.

4 Comparison of different methods to calculate MSB

Squigualiser and *Remora* (formerly *Tombo*) use different methods to determine the MSB for a given k-mer, allowing signal data to be aligned at single-base resolution ('base-shift correction'). The method we developed for *Squigualiser* is outlined in detail here. In contrast, *Remora*'s documentation states that a Kruskal-Wallis H-test statistic method is used, however, further methodological comparison is not possible as there is no detailed description of how this is performed. *Uncalled4* determines the 'central base' in a k-mer (similar to *Squigualiser*'s MSB) by computing its "substitution profile": the average normalised current change observed by substituting each base at each k-mer position. This is an alternative method to the approach we have developed, but appears to be similarly valid.

Aside from this methodological difference, a key advantage of *Squigualiser* is that the tool can automatically calculate the MSB and appropriate base-shift correction for an unseen user dataset. As the MSB differs for different protocols (ONT pore type, DNA vs RNA, signal alignment method, etc), this is critical for optimal visualisation on alternative datasets. While *Uncalled4* includes a method for base-shift correction (described above) this is not automatically calculated and we cannot see instructions on how the user can calculate it for themself, on a given dataset. Table 3 compares MSB methods between *Remora* (*Tombo*), *Uncalled4* and *Squigualiser*

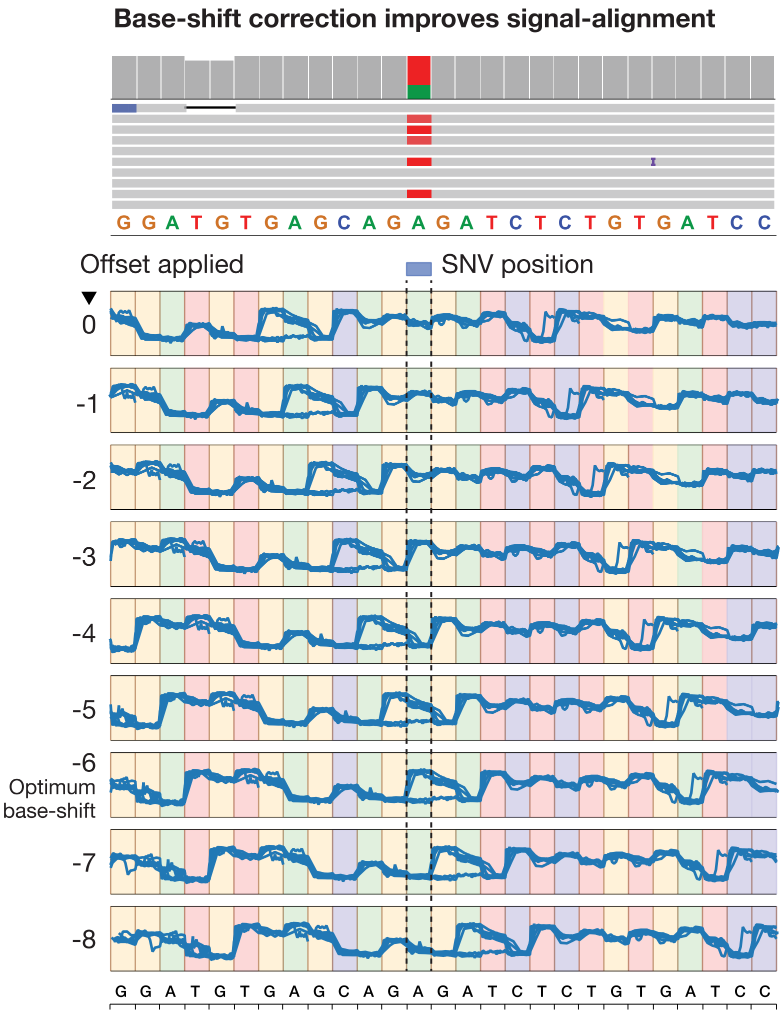

Figure 11: **Evaluating *Squigualiser* k-mer-to-base shift correction.** The k-mer-to-base shift correction strategy is designed to optimise signal alignment visualisation by correcting the arbitrary offset between a given nucleotide and its most relevant corresponding event in aligned signal. *Squigualiser* calculates and corrects this offset for a given protocol/dataset; for the dataset displayed here, an offset of -6 was determined by *Squigualiser* to be optimum. To test whether this correction led to improved resolution, we inspected known heterozygous A-to-T SNVs, reasoning that these sites should show bimodal signal values corresponding to the reference and alternate alleles, and that the optimum offset should show the highest degree of bimodality. The upper track shows basecalled read alignments to a single example SNV displayed in the IGV genome browser. The lower tracks show signal-alignment pileup images from *Squigualiser* for the same set of reads and the same SNV site, with a range of different offset values applied (0 to -8). The specific SNV example shown has a read-depth of 10.

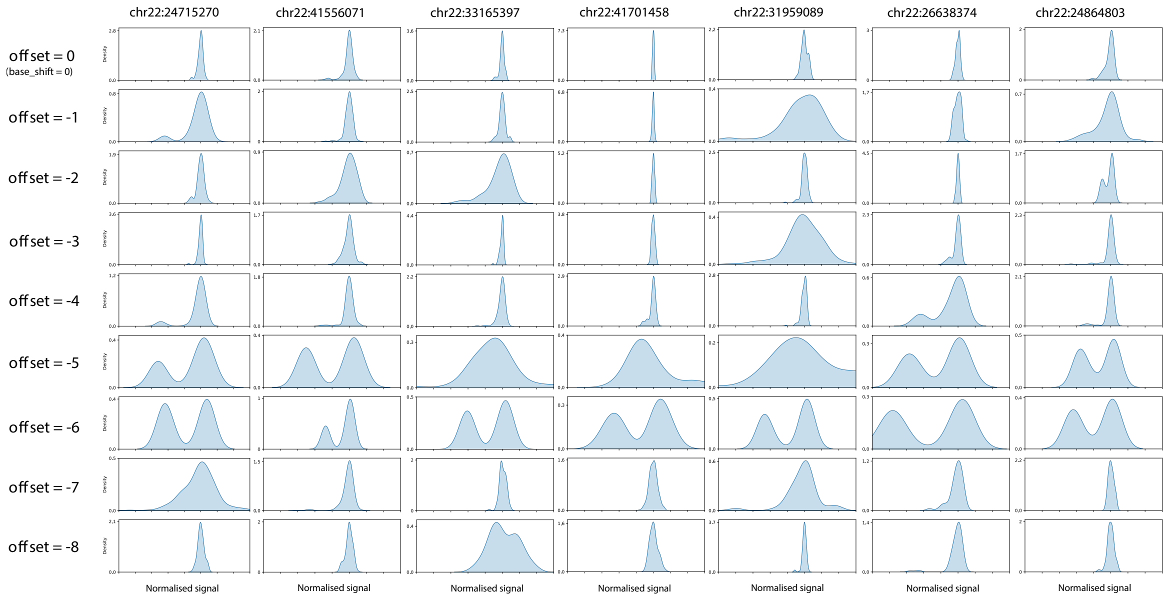

Figure 12: **Evaluating *Squigualiser* k-mer-to-base shift correction.** Kernel density plots show distribution of signal values at a SNV site for n = 7 randomly selected A-to-T SNVs. Each SNV is displayed separately and signal values associated with a given SNV are normalised by dividing each raw value by the mean of all values recorded for that SNV. For each SNV, the optimum offset of -6 calculated by *Squigualiser* is associated with the highest degree of bimodality. The DNA sequencing dataset has 15-fold mean sequencing depth.
