# Supplementary material for: Interactive visualization of nanopore sequencing signal data with Squigualiser
Source: Bioinformatics. 2024 Aug 13;40(8):btae501. doi: 10.1093/bioinformatics/btae501 (PMC11335371; doi:10.1093/bioinformatics/btae501)
Supplement: btae501_Supplementary_Materials [file btae501_supplementary_materials.zip › SupplementaryMaterials/Supplementary Note 4.pdf]

Supplementary Note 4: Visualising a genomic region that has a CpG methylated site

Hiruna Samarakoon, Kisaru Liyanage, James M. Ferguson, Sri Parameswaran,
Hasindu Gamaarachchi, Ira W. Deveson

July 1, 2024

Methylated cytosine bases cause differences in the nanopore current level compared to non-methylated bases. This document explains how such current differences can be observed using a squigualiser pileup plot. To visualise 5mC DNA modifications, we processed a recent DNA sequencing dataset (R.10.4.1, LSK114 SRA: SRR23215366) from the genome reference sample HG002 with Squigualiser. The dataset was basecalled (Guppy), aligned to the human reference genome (hg38; minimap2), and 5mC modifications were detected with F5c call-methylation. In parallel, we performed signal alignment with F5c eventalign and generated signal-to-reference data plots with Squigualiser.

The steps to visualise a region with a half-methylated CpG site that effectively demonstrates differences in current levels are given below.

1. Filter a genomic site from the methylation frequency file where the frequency is approximately 0.5.
2. Filter the read ids from the call-methylation TSV file where the log likelihood ratio is less than -2 and above 2 for unmethylated and methylated reads, respectively.
3. Use the filtered read ids to create a pileup plot:

```
squigualiser plot_pileup [OPTIONS] -l readlist -f reference.fa -s reads.blow5 -a
    eventalign.bam --region region
```

Visual inspection highlights the distinct signal pattern between methylated vs unmethylated reads aligned to CpG sites, assisting the user to validate/interpret the meth-calling results from F5c. In this example, we considered the $+$ strand reads covering the site chr1:92790687 (1-based coordinate) with a methylation frequency of 0.542. As shown in the overlap plot in Fig.1 (top track), the site C38 (marked by red block) has two distinct current levels. The higher current level corresponded with the unmethylated read_ids in the methylation calls table (Table 1). The lower current level corresponded with the methylated read_ids. Out of the 8 total reads in the pileup, 3 were unmethylated, and 5 were methylated.

Table 1: Methylation calls from *f5c* call-methylation. Note that positions have been manually edited to be 1-based to match with the coordinate convention of squigualiser.

Chromosome	Strand	Position	Read Name	Log Lkhd Ratio	Log Lkhd Methylated	Log Lkhd Unmethylated	No. of Calling Strands	No. of Motifs
chr1	+	92790687	d2d2c018-f49f-436b-a038-e441f95a49ad	-2.69	-162.08	-159.39	1	1
chr1	+	92790687	5ae057b3-cde0-4248-a905-d18a75079ba2	4.6	-127.22	-131.82	1	1
chr1	+	92790687	caad6a89-8ad5-485d-9ed9-903e99ed1b6a	3.8	-196.16	-199.95	1	1
chr1	+	92790687	0aa2c317-d8c4-4861-ac6f-87bab7fff2d8	4.77	-134.04	-138.81	1	1
chr1	+	92790687	fdb21051-2388-446d-acf2-a6848669ced8	2.62	-84.27	-86.89	1	1
chr1	+	92790687	1e12424f-dcab-4cd0-83c3-79afc1326199	-4.41	-120.27	-115.86	1	1
chr1	+	92790687	362dc957-024e-4c56-a26a-c7fe4cd07304	4.15	-198.4	-202.56	1	1
chr1	+	92790687	6b960395-8f61-4b8f-880d-e1852a5556a8	-2.78	-147.48	-144.7	1	1

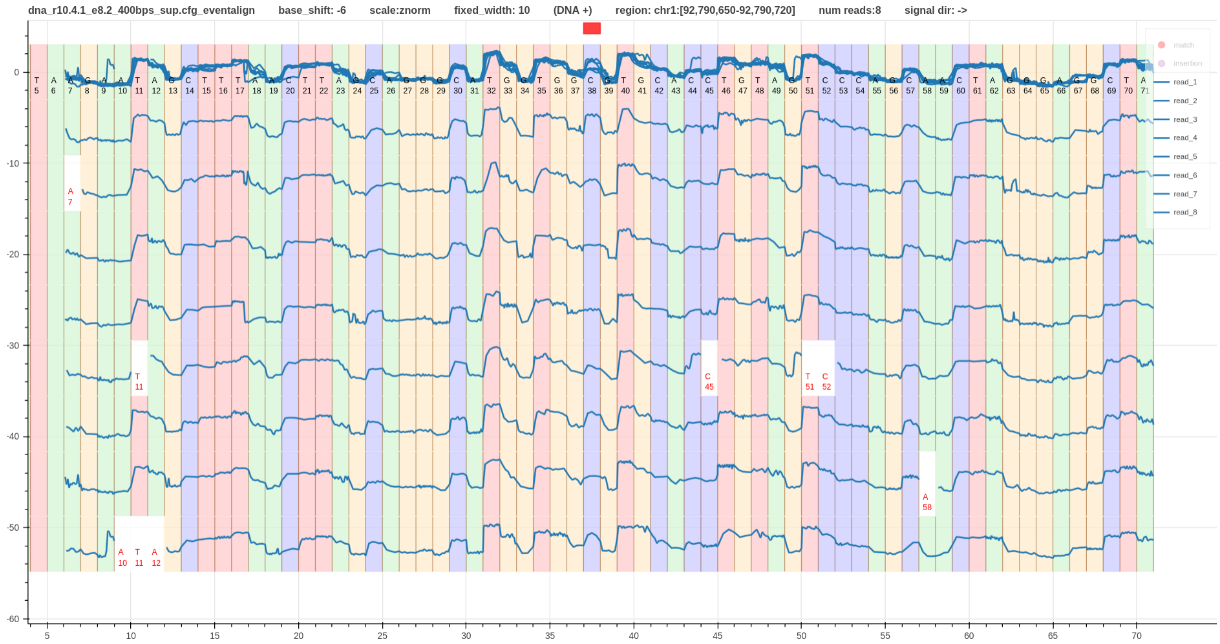

Figure 1: *Squigualiser pileup* plot including the overlap of the signals at the top. The methylation detection conducted using in *f5c* indicated that the site C38 has a methylation frequency of 0.542.
