# Supplementary material for: Interactive visualization of nanopore sequencing signal data with Squigualiser
Source: Bioinformatics. 2024 Aug 13;40(8):btae501. doi: 10.1093/bioinformatics/btae501 (PMC11335371; doi:10.1093/bioinformatics/btae501)
Supplement: btae501_Supplementary_Materials [file btae501_supplementary_materials.zip › SupplementaryMaterials/Supplementary Note 5.pdf]

# Supplementary Note 5: Visualising RNA modifications

Hiruna Samarakoon, Kisaru Liyanage, James M. Ferguson, Sri Parameswaran, Hasindu Gamaarachchi, Ira W. Deveson

July 1, 2024

In summary, in this document, we inspect synthetic direct RNA data from ONT rna_r9.4.1_70bps chemistry. To visualise pseudouridine ($\Psi$) RNA modifications, we downloaded publicly available direct RNA sequencing data generated from synthetic RNA molecules produced with ($\Psi+$) and without ($\Psi$-) modifications in place of canonical U bases. The dataset was basecalled (Guppy), aligned to the relevant synthetic reference sequences (minimap2), then signal alignments were generated with F5c eventalign and visualised with Squigualiser. Visual inspection showed poor alignment quality for the $\Psi+$ data, with many gaps appearing in regions where T(U) bases appear in the reference sequence. By retrieving signal values at these sites, we were able to create a customised ONT k-mer pore model that includes expected signal values for RNA k-mers containing $\Psi$ modifications, and providing this pore model to F5c eventalign led to major improvements in signal alignment quality, with many fewer gaps observed in the $\Psi+$ data. This approach enabled visual comparison of aligned signal values at known $\Psi+$ vs $\Psi$- sites, assisting the user to identify the effects of this RNA modification on the underlying signal.

Table 1: Dataset Information

| Dataset name | SRA accession description | Preparation |
|---|---|---|
| unmodified (UnMod_37C) | direct RNA-seq of GFP mRNA (SRR22888949) | synthetic RNA with all sites unmodified |
| modifiedU (Mod_37C) | direct RNA-seq of GFP mRNA (SRR22888950) | synthetic RNA with all Us modified |

The following steps were executed.

1. Basecall the two datasets using guppy_v6.3.7 with the model rna_r9.4.1_70bps_hac_prom.cfg.
2. Align the reads to the synthetic reference using *minimap2*.
3. Generate a signal-to-reference alignment using *squigualiser realign* program (see **Supplementary Note 2**).
4. Generate another signal-to-reference alignment using *Nanopolish/f5c eventalign*.
5. Evaluate signal-to-reference alignments by visualising the signal pileups (using *Squigualiser plot_pileup*).
6. Improve *Nanopolish/f5c* signal-to-reference alignment by training the k-mer model using *Nanopolish train* program.
7. Evaluate the efficiacy of *Nanopolish training* by observing the signal-to-reference alignments generated using the k-mer models after each training round.
8. Observe the feature differences in the unmodified and modified signals.

When using the default basecalling parameters, the reported pass read percentages were 92% for the unmodified dataset and 62% for the modifiedU dataset. The very low pass percentage (62%) for the modifiedU dataset indicates that the synthetically modified U has very different signal features than the normal U. This effect has made the basecalling models erroneous around U bases.

Table 2: *Minimap2* alignment statistics

| | minimap2 params | | Reads | | | | Bases | | | | |
|---|---|---|---|---|---|---|---|---|---|---|---|
| Dataset | -K (k-mer size) | -s (DP score) | Total | Mapped | Unmapped | Mapped % | Total | Mapped | Mapped % | Mismatches | Error rate |
| unmodified | default (15) | default (80) | 4622 | 4615 | 7 | 0.998 | 5137565 | 4922590 | 0.959 | 631666 | 0.129 |
| modifiedU | default (15) | default (80) | 445310 | 238402 | 206908 | 0.5358 | 459273108 | 197679705 | 0.431 | 54221392 | 0.275 |
| modifiedU | 10 | 50 | 445310 | 432604 | 12706 | 0.9718 | 459273108 | 347414498 | 0.757 | 97745894 | 0.282 |

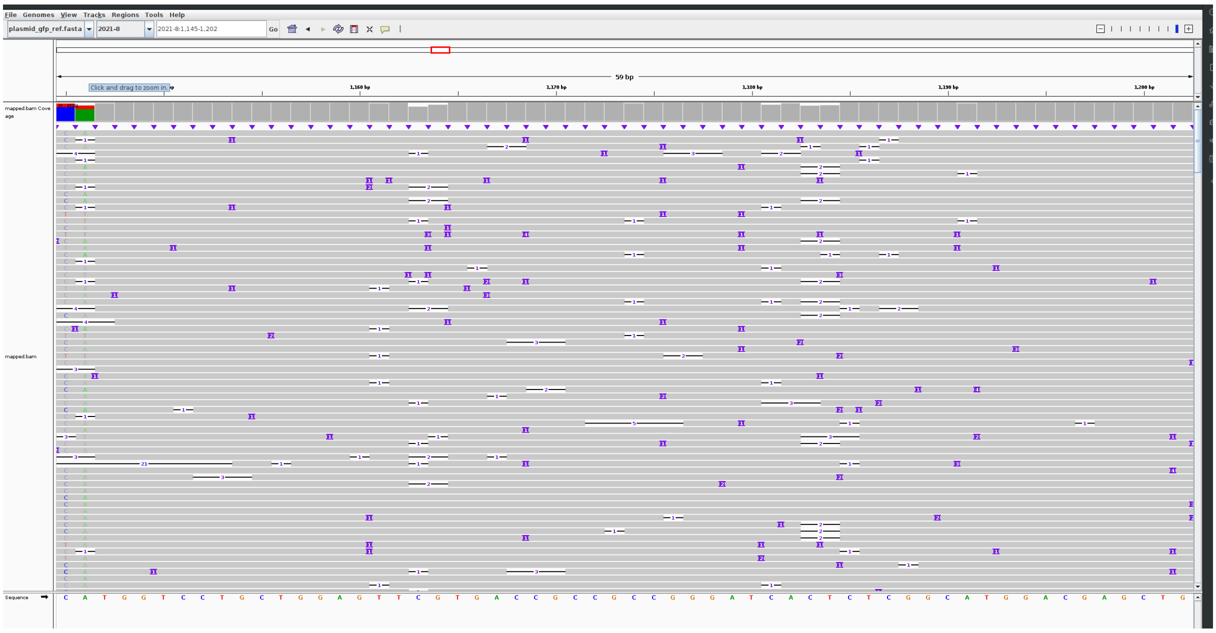


Figure 1: Unmodified read-to-reference alignment

This can be further confirmed by looking at the metrics after aligning the passed reads to the synthetic reference (Table 2). The modifiedU dataset's mapping percentage is very low (53%) compared to the unmodified dataset's mapping percentage (99%). The k-mer size and alignment score parameters were changed in minimap2 to get a better mapping percentage (97%, Table 2).

By comparing the reference-read alignments on IGV, we can observe that the basecaller has basecalled almost all modifiedUs as C (Fig. 1 & 2).

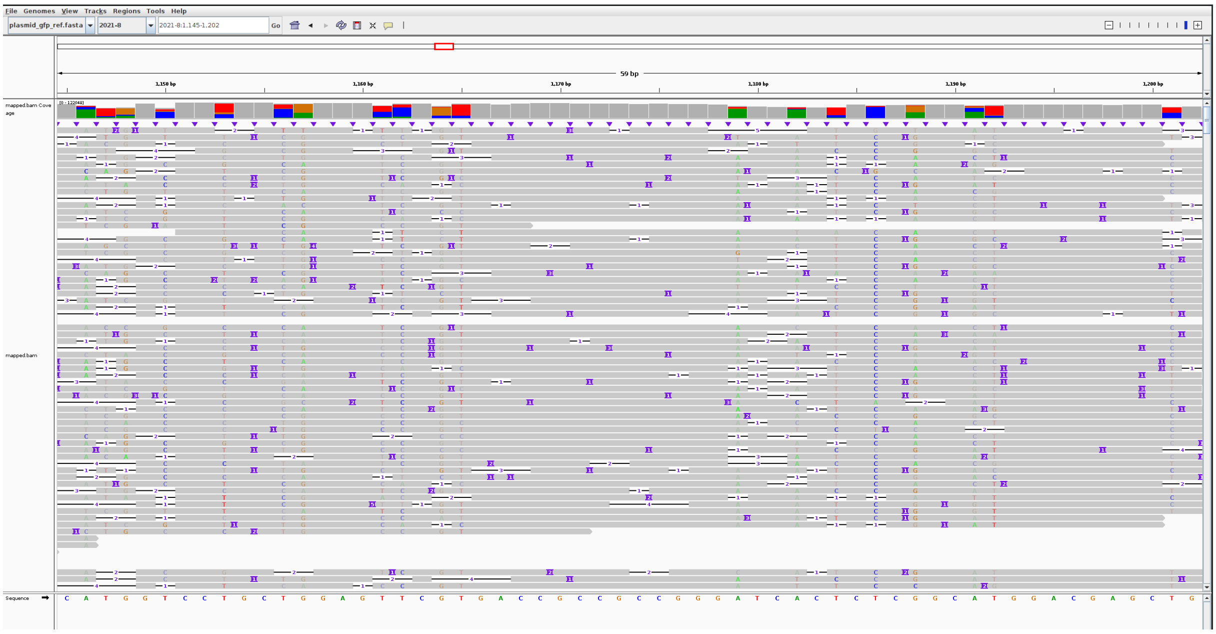

Figure 2: ModifiedU read-to-reference alignment

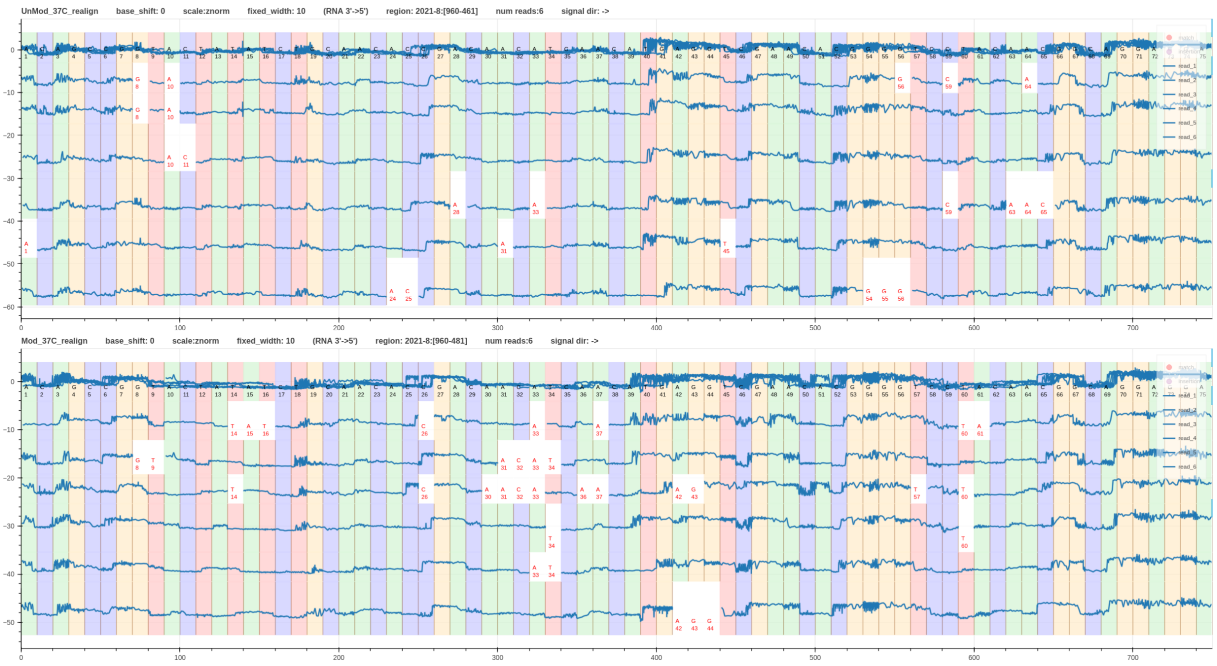


Figure 3: Unmodified and modifiedU signal-to-reference alignment using *Squigualiser realign*

Now let's proceed to the signal alignment stage.

*Squigualiser realign* method appear to be robust around modified Us as it uses the basecaller's move table (it does not matter if it is a C or U as long as it is a counted as a move). However, the boundaries of signal alignment are not refined in this method (Fig. 3).

Fig. 4 shows a comparison between the unmodified and modifiedU data signal alignment using *Nanopolish/f5c eventalign*. The signal alignment is erroneous around U bases in the modifiedU data. K-mers with modified Us have different current levels than the k-mers with unmodified Us. Since *Nanopolish/f5c eventalign* relies on k-mer model, it is critical that the k-mer model has the updated current levels for the modified k-mers. Therefore, *Nanopolish train* program was used to train a k-mer model for the modified data.

The *Nanopolish* training was carried out for 10 rounds. The trained k-model at each round was given as a custom model to *f5c* eventalign program to align the modifiedU signal data. However, it was observed that the signal alignment accuracy did not improve after the first two rounds (Fig. 5). Hence, the k-model trained in round 1 (0-based) was used to align the modifiedU data (Fig. 6).

```
f5c eventalign -b minimap2.bam -r reads.fastq -f genome.fa -s reads.blow5 -a eventalign.
    bam --kmer-model roundX.model
```

Fig. 7 takes a closer look at the current level differences of the k-mers with U between unmodified and modifiedeU datasets. We observe that the current level of a k-mers with modified U drops below the current level of the corresponding unmodified k-mer. This observation is more pronounced when there are several modified Us together.

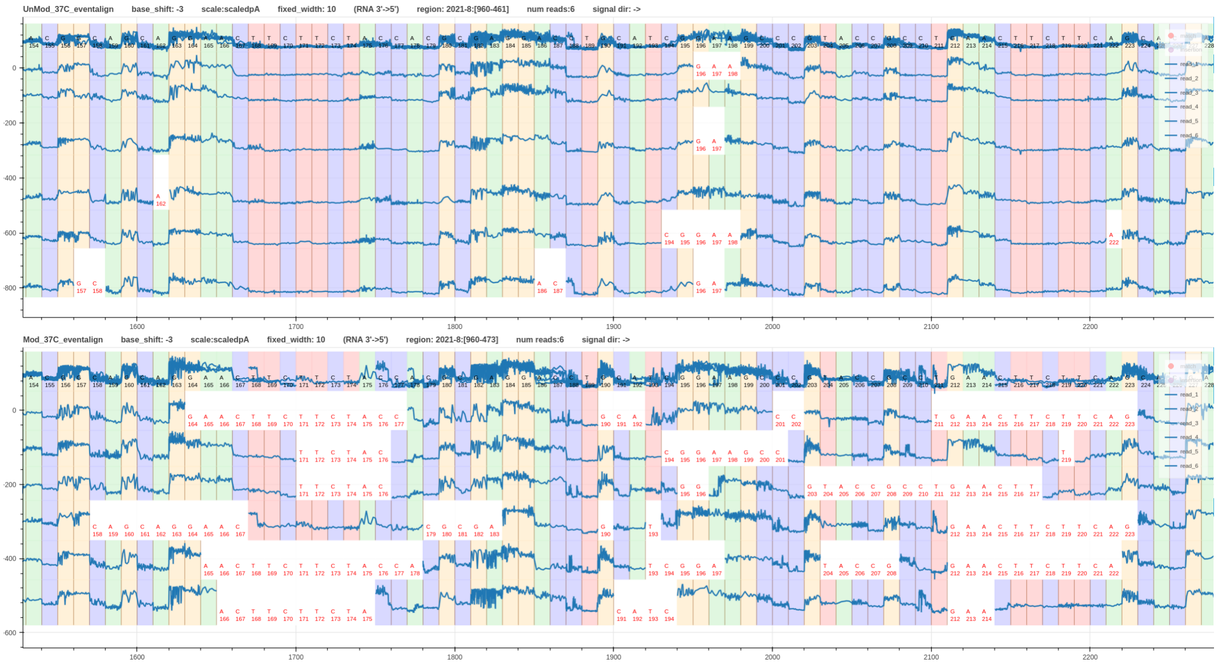


Figure 4: Unmodified (top pileup track) and modifiedU (bottom pileup track) signal-to-reference alignment using *Nanopolish/f5c eventalign*

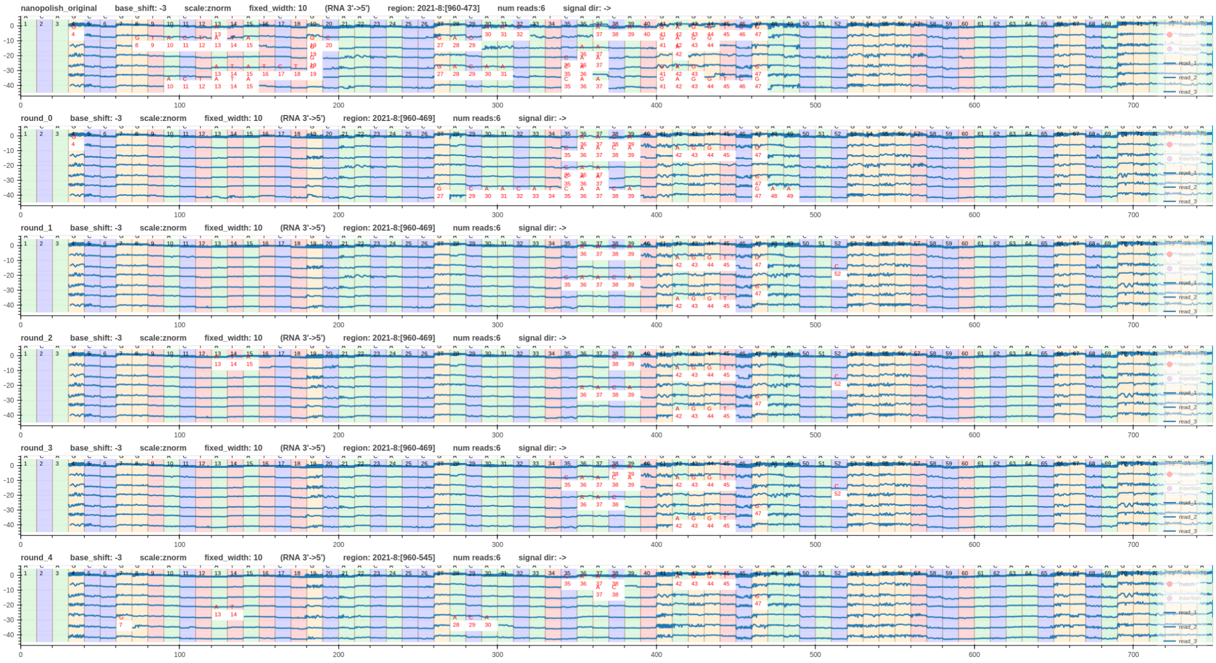


Figure 5: ModifiedU signal-to-reference alignment using *Nanopolish/f5c eventalign* with trained k-mer models. The topmost pileup track is for the original k-mer model and the subsequent tracks are for trained models from training rounds 0 to 4.

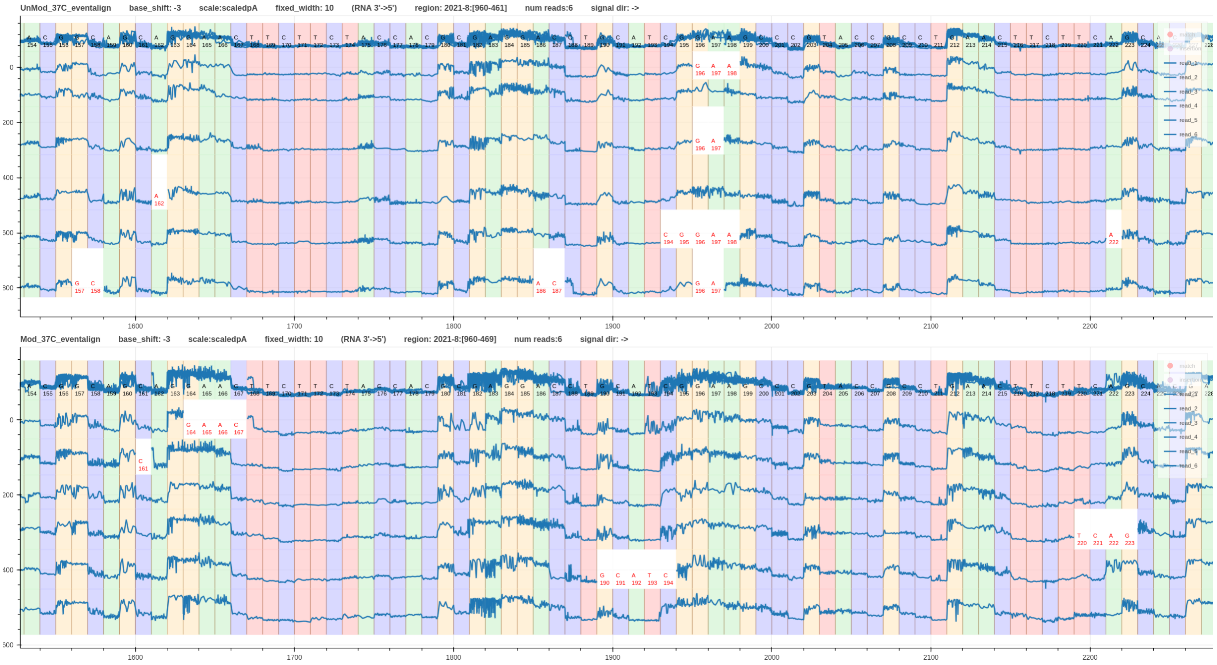


Figure 6: Unmodified signal-to-reference alignment with original k-mer model (top pileup track) and modifiedU signal-to-reference alignment with trained k-mer model (bottom pileup plot). Alignment performed using *Nanopolish/f5c eventalign*.

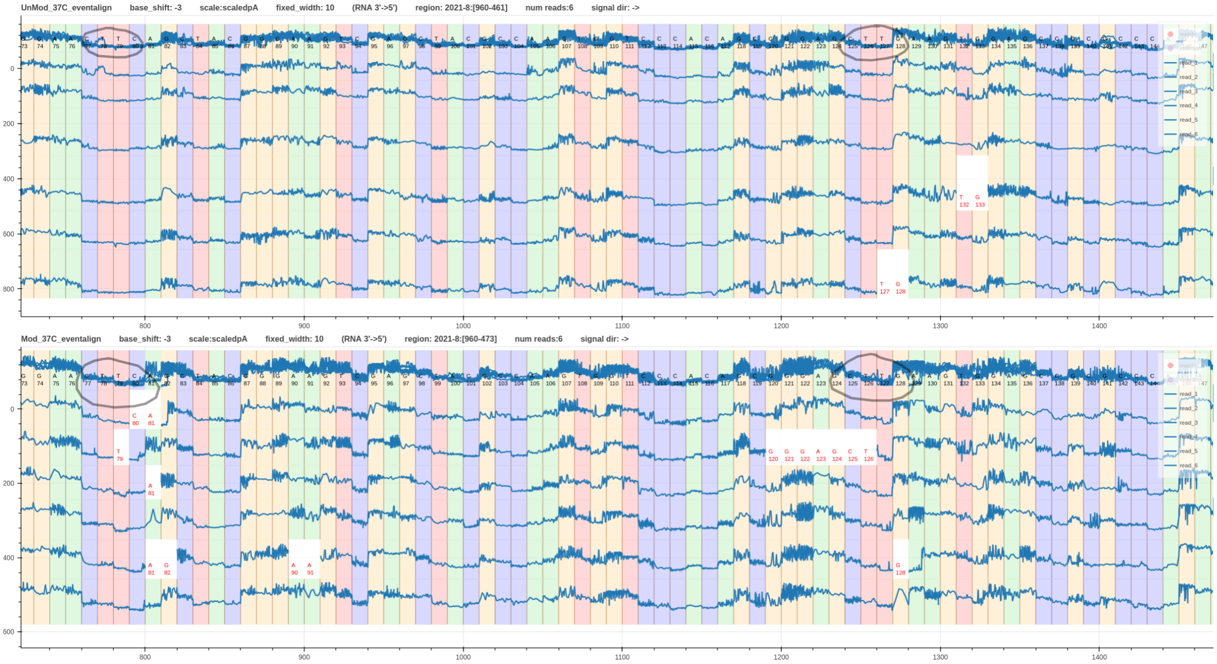


Figure 7: Unmodified and modifiedU current level comparison
