# Supplementary material for: Interactive visualization of nanopore sequencing signal data with Squigualiser
Source: Bioinformatics. 2024 Aug 13;40(8):btae501. doi: 10.1093/bioinformatics/btae501 (PMC11335371; doi:10.1093/bioinformatics/btae501)
Supplement: btae501_Supplementary_Materials [file btae501_supplementary_materials.zip › SupplementaryMaterials/Supplementary_File_1.html]

dna\_r10.4.1\_e8.2\_400bps\_sup.cfg\_evligned\_vs\_sim
